# Image processing with Optical matrix vector multipliers implemented for encoding and decoding tasks

**DOI:** 10.1038/s41377-025-01904-z

**Published:** 2025-07-22

**Authors:** Minjoo Kim, Yelim Kim, Won Il Park

**Affiliations:** Division of Materials Science and Engineering, Hanyang University, Seoul, Republic of Korea

**Keywords:** Imaging and sensing, Optoelectronic devices and components

## Abstract

This study introduces an optical neural network (ONN)-based autoencoder for efficient image processing, utilizing specialized optical matrix-vector multipliers for both encoding and decoding tasks. To address the challenges in efficient decoding, we propose a method that optimizes output processing through scalar multiplications, enhancing performance in generating higher-dimensional outputs. By employing on-system iterative tuning, we mitigate hardware imperfections and noise, progressively improving image reconstruction accuracy to near-digital quality. Furthermore, our approach supports noise reduction and optical image generation, enabling models such as denoising autoencoders, variational autoencoders, and generative adversarial networks. Our results demonstrate that ONN-based systems have the potential to surpass the energy efficiency of traditional electronic systems, enabling real-time, low-power image processing in applications such as medical imaging, autonomous vehicles, and edge computing.

## Introduction

Optical image processing is fundamental to applications in autonomous navigation, security, defense, and medical diagnostics^1–4^. It begins with capturing images using cameras and converting these analog visuals into digital data, followed by image recognition, reconstruction, interpretation, and decision-making. However, digital images are typically large and contain extraneous information, such as background patterns and noise, necessitating preprocessing to reduce dimensionality while retaining essential information. Image encoders, including autoencoders—a popular deep neural network (DNN) architecture—transform high-dimensional input into a compact latent space by compressing the data through a series of layers^5–9^.

Autoencoders also employ decoder networks to reverse this process, reconstructing the compressed data back to its original form^10–12^. Decoders are trained to closely reproduce the original input, which, combined with encoder networks, makes autoencoders valuable for tasks like data compression, noise reduction, and feature extraction. In generative models, such as variational autoencoders (VAEs) and generative adversarial networks (GANs), decoders generate realistic and contextually appropriate images, crucial for image enhancement, data augmentation, and creative applications in art and film^13–15^.

Despite significant advancements in computational efficiency, driven by both software and hardware improvements^16,17^, the electronic processing of DNNs using accelerators faces challenges such as high energy consumption and limitations in scaling parallelism and speed efficiency. Optical neural networks (ONNs) offer an alternative with effective operations such as matrix-vector multiplication (MVM) and nonlinear activation of optical signals^8,18–23^. Fully optical multilayer, nonlinear pre-processor for 2D image processing has been implemented using components like spatial light modulators (SLMs), liquid crystal displays (LCDs), diffraction optical elements (DOEs), nonlinear optical materials, and saturable amplifier (e.g., image intensifier), enabling ONNs to efficiently compress and process large images with lower latency^5,24–27^.

Current ONN systems have shown superior performance in encoding operation, using optical analog computing to compress image data before digital conversion^19,28–30^. However, decoding requires a similar computational resource, highlighting the need for advancements in both encoding and decoding. Given the ultimate ONN goal is to implement multi-layer neural networks primarily through optical methods–or at least in combination with analog approaches^5,6^–developing effective optical decoders is crucial to fully utilize the potentials of autoencoders in handling complex real-world visual data. Although research has been conducted on 2D image encoding and decoding using diffractive deep neural networks (D2NNs)^31,32^, these systems inherently require coherent light as input. This presents a fundamental limitation because actual incoherent image inputs must undergo an optoelectronic conversion process to generate coherent input light. This additional step introduces complexity and potential latency, undermining the benefits of optical processing. Direct encoding of natural images^5,33^ and subsequent decoding with high accuracy and low latency are essential for applications like autonomous navigation, medical diagnostics, and defense, where fast and reliable image processing directly impacts safety and performance.

In this work, we present an ONN-based autoencoder that leverages optical matrix-vector multipliers for both encoding and decoding tasks. While previous MVM strategies efficiently handle scenarios where input dimensions exceed output dimensions^5,31,33–36^, they exhibit limitations when dealing with larger output sizes. To overcome this, we propose a method for optimizing output processing via scalar multiplications, enhancing the efficiency of higher-dimensional output generation. We introduced on-system iterative tuning to mitigate hardware imperfections and noise, which gradually improved image reconstruction accuracy, nearing the quality of digital processors. Additionally, we extended our approach to noise reduction and optical image generation, enabling functions such as denoising autoencoder (DAE), VAE, and GAN. Our analysis indicates that, with further optimizations, ONN-based autoencoders have the potential to significantly exceed the energy efficiency of electronic systems. These advancements could lead to fully optical implementations capable of near-instantaneous processing, substantial energy savings, and real-time handling of complex tasks.

## Results

### Implementation of optical matrix-vector multipliers for encoding and decoding

**Fig. 1:**
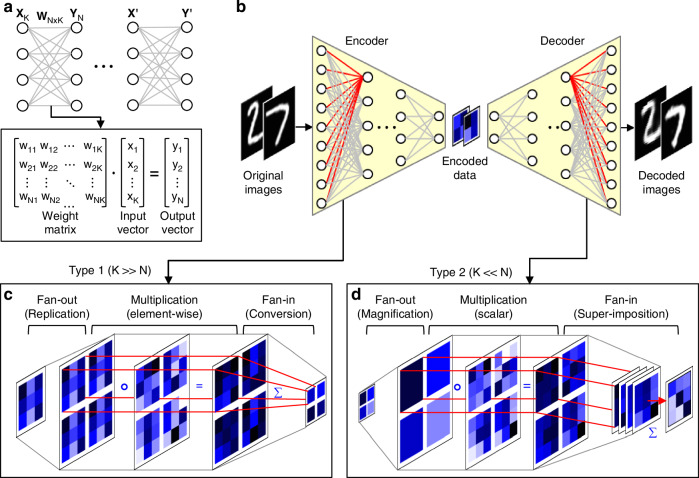
**Schematic of the optical MVM operations used in the ONN-based autoencoder**. **a** General structure of a DNN utilizing FC-NNs with MVMs. The key operation, MVM, involves multiplying a weight matrix *W*_N×K_ by an input vector *X*_K_, performing element-wise multiplications and accumulating the results, producing an output vector *Y*_N_. **b** Representation of an autoencoder built with FC-NNs, where the encoder compresses high-dimensional input data into a lower-dimensional latent vector, and the decoder reconstructs this data. **c** Type 1 MVM operation showing the fan-out, multiplication, and fan-in processes for encoding tasks where the output dimensions are smaller than input dimensions (*K* >> *N*). **d** Type 2 MVM operation showing the enhanced strategy for decoding tasks, where the output dimensions exceed input dimensions (*K* << *N*)

Figure 1a schematically illustrates a general DNN composed of fully connected neural networks (FC-NNs). The key operation for computing a DNN inference layer, MVM, is shown in the lower boxed panel in Fig. 1a. MVM involves multiplying a weight matrix **W** by an input data vector **X**, performing parallel multiply-accumulate (MAC) operations to produce an output vector **Y** (i.e., ${{\bf{Y}}}_{{\rm{N}}}={{\bf{W}}}_{{\rm{N}}\times {\rm{K}}}\cdot {{\bf{X}}}_{{\rm{K}}}$). This output is modulated by a nonlinear activation function *f* and passed to the next layer.

In an autoencoder built with FC-NNs (Fig. 1b), the encoder compresses high-dimensional input data into a lower-dimensional latent vector through multiple layers of MVM followed by nonlinear activations. The decoder reverses this process, aiming to reconstruct the original data as accurately as possible. Most computational resources in autoencoders are consumed by MVM operations, particularly in the first and last layers of the encoder and decoder networks.

Recent advancements in ONNs have explored various methods for implementing MVM^35,37–40^. Optical matrix-vector multipliers specifically designed to directly process natural 2D images have recently been developed, sharing several common features (these methods and systems are referred to as Type 1 MVM and multiplier)^5,33–36^. Figure 1c illustrates the key steps using a simple input vector (*K* = 9 elements in 3 × 3 pixels) and output vector (*N* = 4 elements in 2 × 2 pixels):

1. Fan-out: The 2D input image block is copied into an 2 × 2 array, which can be optically implemented using a micro-lens array or DOE^5,34^.
2. Multiplication: Element-wise multiplication is achieved by projecting the input image copies onto weight matrix patterns on an SLM or LCD, with the weight matrix represented as an array matching the dimensions of fan-out input images.
3. Fan-in: Summation of the intensity-modulated light components in each block is realized by focusing them onto a detector array.

This operation involves parallel vector-vector-dot products between input and weight vectors, with the first one, ${{\rm{y}}}_{1}={{\bf{W}}}_{1{\rm{K}}}\cdot {{\bf{X}}}_{{\rm{K}}}$ (where **W**_1K_ represents first-row weight vectors with K elements), highlighted by red lines in Fig. 1c and corresponding to the shaded components in the matrix operations shown in Supplementary Fig. S1a. This method is efficient for tasks like encoding, where output dimensions are smaller than input dimensions (i.e., K>>N), as it can be implemented using a small number of lenses and detectors for optical fan-out and fan-in, respectively.

However, Type 1 multiplier is inefficient for decoding operations where output dimensions exceed input dimensions (i.e., K<<N) due to increased complexity in optical implementations of fan-out and fan-in processes. Instead, we propose a Type 2 MVM operation strategy, as illustrated in Fig. 1d, using an input vector (4 elements in 2 × 2 pixels) and output vector (9 elements in 3 × 3 pixels). This strategy modifies the three key steps:

1. Fan-out: Instead of replicating, each pixel of the input is magnified.
2. Multiplication: The magnified input pixels are projected onto a weight matrix encoded as a 2 × 2 array of blocks, where each block corresponds to a column vector in the weight matrix (unlike Type 1, where it represents a row vector). This allows parallel scalar multiplications, producing an output with the same dimension as the weight matrix. The red lines in Fig. 1d represent the first of scalar multiplications, corresponding to the red-shaded components in the matrix operations in Supplementary Fig. S1b.
3. Fan-in: Element-wise summation is achieved by overlapping all blocks from the previous step.

### Simulation analysis of optical MVM strategies for decoding performance

**Fig. 2:**
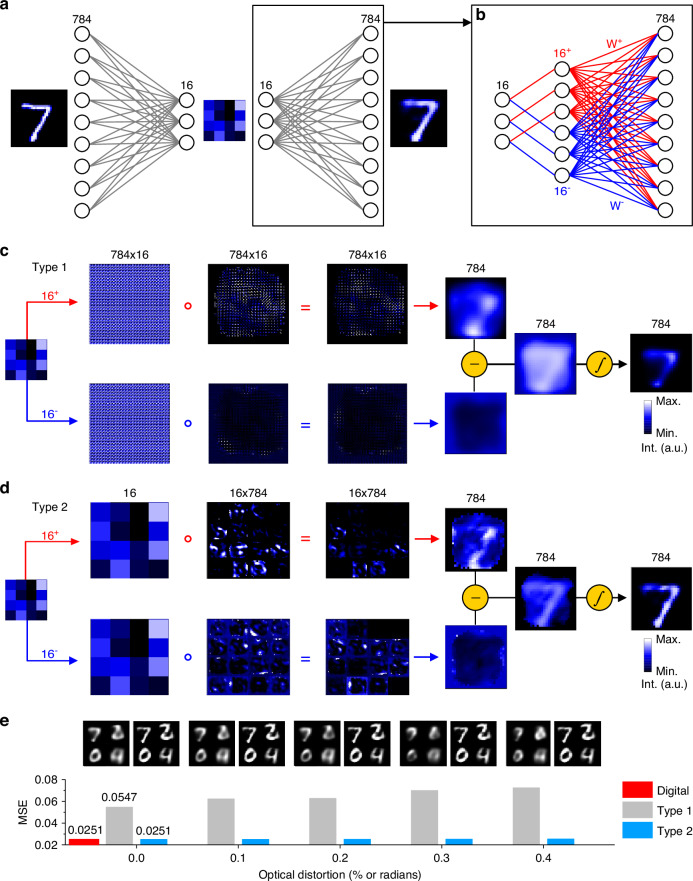
**Simulation results comparing the performance of Type 1 and Type 2 multipliers in decoding tasks**. **a** Autoencoder architecture trained on the MNIST dataset with 784 input/output neurons and 16 encoding neurons. **b** Weight matrix handling both positive and negative elements for optimal decoding results. **c**, **d** Visual comparison of the fan-out input patterns, weight matrix patterns, post-multiplication patterns, and fan-in images (top panels for positive weights and bottom panels for negative weights), followed by subtraction between the pairs and sigmoid activation to reconstruct a digit “7” using Type 1 and Type 2 multipliers. **e** MSE loss comparison between digital, Type 1, and Type 2 multipliers under varying optical distortion levels. Top panels compare the corresponding reconstructed results. Optical distortion is quantified using vertical and lateral shifts (random displacements within ±X% of the image width, modeled as a uniform distribution) and shear transformations (angular distortions within ±X radians). See Supplementary Fig. S3 for schematic illustrations of these distortions

To evaluate the effectiveness of Type 2 MVM for decoding, we simulated 2D image reconstruction from compressed data using both types of multipliers. An autoencoder with 784 input/output neurons and 16 encoding neurons was trained on the MNIST train dataset (Fig. 2a), and the trained weights were transferred to our model for simulation. The decoder network used a pair of weight matrix (784 × 16) to handle positive and negative elements separately (red and blue lines in Fig. 2b). While clamping the encoder weights to positive values caused minimal degradation^5,34^, applying this constraint to the decoder significantly reduced performance (Supplementary Fig. S2), necessitating the use of both positive and negative weights for optimal results.

Figure 2c, d compares the simulated results for Type 1 and Type 2 multipliers, displaying the fan-out input patterns, weight matrix patterns, post-multiplication patterns, and fan-in images for an MNIST image of the digit “7”. The 784-pixel image pairs are subtracted and processed with a sigmoid activation to complete the decoding process.

These approaches were applied to 3000 MNIST test images to evaluate and compare the mean square error (MSE) of the Type 1 and Type 2 multipliers. The MSE values were calculated using pixel intensity values normalized to the range of 0 to 1. The Type 1 multiplier exhibited an average MSE loss of 0.0547, which was higher than the 0.0251 loss achieved by both the digital and Type 2 multipliers, as shown in Fig. 2e. This discrepancy primarily arises from the optical fan-in process, where 784 × 16 pixel images (or 16 × 784 for Type 2) are reduced to 784 pixels. In the case of the Type 1 multiplier, summing each 4 × 4 pixel block is required to produce a single pixel value for each of the 784 output vectors. When implemented optically during image capture, this downscaling introduces crosstalk between adjacent pixels, leading to additional reconstruction loss.

To model this effect, simulations applied an image reduction method, downsampling a 112 × 112 pixel image to a 28 × 28 pixel image using interpolation. This mimicked the challenges of optical fan-in, such as pixel crosstalk and reconstruction loss. By contrast, the Type 2 multiplier avoids this issue by employing image overlap to achieve element-wise summation. This method bypasses the need for pixel block reduction and replicates digital computations more effectively, resulting in lower reconstruction loss.

Additional losses in practical optical implementations can also arise from optical distortions, such as shearing or misalignment between images. These factors are particularly impactful for the Type 1 multiplier during element-wise multiplication, where the fan-out input pattern overlaps with the weight matrix displayed on the LCD. Simulations reveal that a distortion of 0.4% increases the MSE loss by 0.073% for Type 1. In contrast, the Type 2 multiplier is less affected by such distortions, as scalar multiplications are not influenced by pixel alignment. The majority of distortions in Type 2 occur in the final fan-in step, and even with a 0.4% distortion, the loss increase remains within 0.025%, highlighting the superior robustness of the Type 2 method for decoding operations.

### Implementation and evaluation of an ONN-autoencoder using Type 1 and Type 2 multipliers

**Fig. 3:**
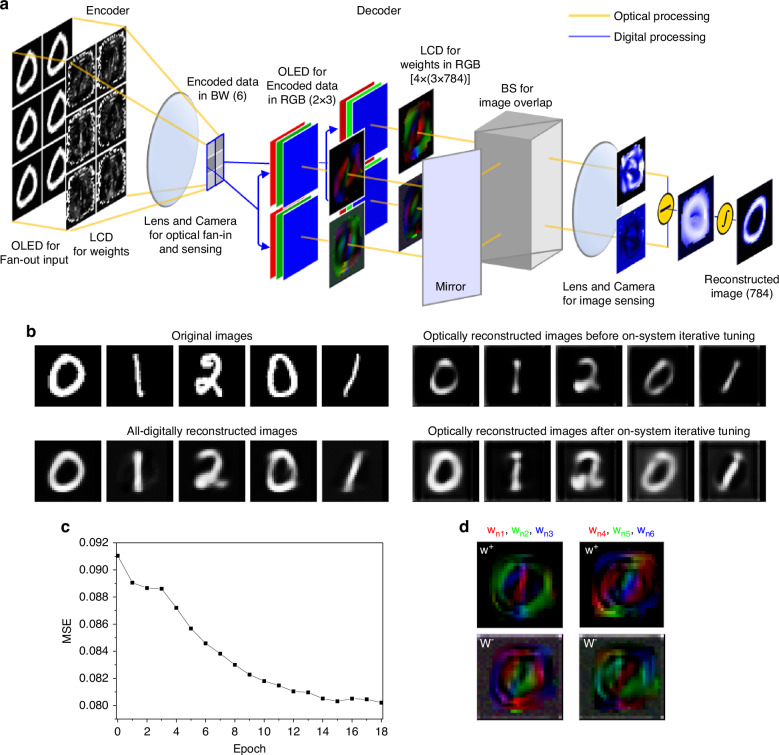
**Hardware implementation and evaluation of the ONN-based autoencoder**. **a** Schematic representation of the autoencoder with an ED of 6, showing the optical setup for the encoder and decoder, utilizing Type 1 and Type 2 multipliers, respectively. In the encoder, weights are clamped to positive values, while in the decoder, weights can be either positive or negative, implemented using pairs of weight patterns. **b** Examples of original images, digitally reconstructed images, and those reconstructed using the optical autoencoder before (epoch 0) and after on-system iterative tuning (epoch 18). **c** Improvement in MSE loss through on-system iterative tuning. **d** Visualization of fine-tuned weights in RGB, where the top and bottom panels represent the positive and negative weight elements, respectively

Based on our analysis of optical MVM methods, we implemented an ONN-autoencoder using a Type 1 multiplier for the encoder network^5,35^ and a Type 2 multiplier for the decoder network. Figure 3a illustrates the hardware implementation scheme of the autoencoder, designed with an ED of 6 (see Supplementary Fig. S4 for hardware implementation). The fan-out input and weights are represented by 6 blocks of 28 × 28 pixel monochromatic images, displayed using an organic light-emitting diode (OLED) display and an LCD panel. After passing through the LCD panel, the input images are focused into tiny point beams via optical lenses, and their intensities are measured by a detector array (as shown in the encoder section of Fig. 3a and Supplementary Fig. S4; see Methods for details).

For the decoder, weights include both positive and negative elements to ensure optimal performance. Normally, the Type 2 MVM operation would require 12 blocks of 28 × 28 pixel images to represent these weights in monochromatic channels. To streamline this process, we utilized RGB color channels in both the OLED display and LCD, reducing the requirement to 4 blocks (as shown in the decoder section of Fig. 3a and Supplementary Fig. S4; see Methods for details). These channels represent the encoded data and weights, with the upper and lower blocks in the LCD corresponding to the positive and negative elements of each row vector in the weight matrix. Within each block, color-wise scalar multiplication occurs as the encoded inputs pass through the LCD weights. The resulting images are then combined using a mirror and a beam splitter, producing a pair of 784-pixel images captured by a monochromatic camera. Finally, digital computations, such as subtraction and sigmoid activation, are performed to complete the decoding operation and reconstruct the final image.

In a simplified demonstration with an encoding dimension (ED) of 6, the ONN autoencoder was applied to a subset of the MNIST dataset, specifically using digits 0, 1, and 2. This test illustrates the effectiveness of the proposed optical multipliers. Figure 3b presents examples of the original images alongside their digitally and optically reconstructed counterparts. Although additional weight adjustments were made to correct for variations in light intensity due to color and position (Supplementary Fig. S5), the decoded images using our ONN multipliers initially showed noticeable discrepancies from the originals and those decoded by fully digital processes, particularly with features appearing thinner and blurred. This was reflected in a higher reconstruction loss, with an MSE of 0.091. These performance declines were attributed to hardware imperfections, such as lens aberrations, color interference in OLED images, and misalignment in image overlap^6,33^.

To address these challenges, we implemented on-system iterative adjustments, which involves fine-tuning the weights through an error-backpropagation algorithm^41^, utilizing 3000 training images and 500 distinct images from the test datasets for validation (see Methods and Supplementary Information 1.5 for details). As the iterative adjustments progressed, the MSE gradually decreased (Fig. 3c), leading to improvements in the reconstructed images. Figure 3d displays the fine-tuned weights in RGB.

### Enhanced optical autoencoder performance through increased encoding dimensions

**Fig. 4:**
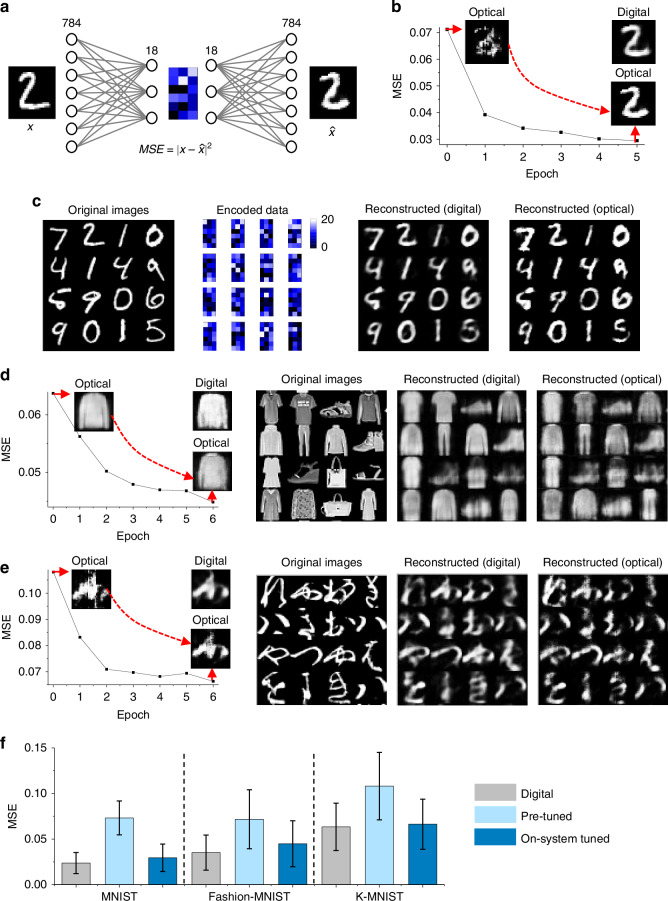
**Enhanced performance of the optical autoencoder by expanding the ED to 18**. **a** Schematic of the expanded autoencoder architecture. **b** MSE loss reduction over iterative tuning epochs, showing improvements in reconstructed image quality for the MNIST dataset. **c** Examples of original test images, encoded data, and reconstructed images, comparing the performance between optical and digital benchmarks. **d**, **e** Application of the autoencoder to more complex datasets, including Fashion-MNIST and K-MNIST, showing reconstruction results from the original inputs after iterative tuning. **f** Comparison of MSE losses for reconstructed images across test datasets, showing performance of the ONN-autoencoder before and after tuning, alongside digital benchmarks. Error bars indicate the standard deviation of MSE values, highlighting variability in reconstruction quality

We further increased the network’s complexity by expanding the ED to 18 (Fig. 4a, see Methods for details). This upgrade improved the optical autoencoder’s ability to handle more complex image reconstruction tasks, including restoring images of all digits from 0 to 9 in the MNIST dataset. However, increasing the ED introduced greater image overlap mismatches during the decoding process, resulting in significant errors at the initial stage. Building on the iterative tuning approach described earlier, we fine-tuned the weights using 3000 training images and validated the result using 500 test datasets (see “Operation of ONN-based Autoencoder” in Supplementary Information for details). This process reduced the loss from 0.0712 at epoch 0 to 0.0295 at epoch 5, where the reconstructed images became significantly clearer, with previously scattered features gradually converging into distinct and recognizable patterns (Fig. 4b).

After tuning, the performance was comparable to the digital benchmark (Fig. 4c), with only a 0.5% increase in MSE loss. Additionally, Fig. 4d, e shows the results of applying the autoencoder to more complex datasets, such as Fashion-MNIST and K-MNIST. Through system fine-tuning, the autoencoder achieved reconstructions closely resembling the digitally-processed or original images, with loss values for Fashion-MNIST and K-MNIST decreasing to 0.045 and 0.066, respectively—comparable to the digital benchmarks of 0.034 and 0.057 (Fig. 4f).

While the mean MSE values showed significant improvement after fine-tuning, the standard deviation of MSE values remained relatively large for both digital and optical reconstructions. This indicates variability in reconstruction quality across individual images, likely stemming from differences in dataset complexity or hardware-related factors such as alignment and light transmission. Representative examples of well-reconstructed images (low MSE), near-average reconstructions, and distorted images (high MSE) are provided in the Supplementary Fig. S6 to illustrate this variability. Despite these challenges, the optical autoencoder demonstrated robustness by consistently reconstructing the majority of images, even from complex datasets, to a quality comparable to digital benchmarks.

Building on these findings, we evaluated reconstruction quality using additional metrics, including peak signal-to-noise ratio (PSNR) and cosine distance, to complement the MSE analysis. As shown in Supplementary Table S1, MSE and PSNR trends demonstrate that the pixel-level fidelity of optical decoding is largely comparable to digital decoding. However, cosine distance revealed the strength of the optical system in preserving global structural patterns, particularly for datasets like Fashion-MNIST and K-MNIST. These datasets, with their intricate patterns, were less affected by pixel-level distortions, resulting in comparable or even better performance for optical decoding in terms of cosine distance. For MNIST, however, the simplicity and high contrast of the images made them more susceptible to optical distortions, favoring digital decoding across all metrics.

Additionally, to evaluate the usability of reconstructed images, we conducted classification tasks using both the original and reconstructed images. A fully connected neural network with a 784-25-10 structure was trained on 60,000 images from the MNIST, Fashion-MNIST, and K-MNIST datasets and evaluated on 3000 unseen test images. The test images included the original images as well as digitally and optically reconstructed images obtained from the network in Fig. 4a.

The results of the classification tasks confirm that the ONN is capable of reconstructing images with sufficient fidelity for use in downstream applications such as object classification (Supplementary Table S2). The high classification accuracy observed with digitally and optically reconstructed images, exceeding 85% for the MNIST dataset, demonstrates that the ONN retains the critical structure and key features of the input images. Although the accuracy for reconstructed images was slightly lower for more intricate datasets, especially for Fashion-MNIST, this is likely due to the relatively simple neural network architecture used in the experimental setup. These findings highlight the robustness of the ONN and suggest avenues for further improvement, such as employing more advanced architectures and optimizing system components.

**Fig. 5:**
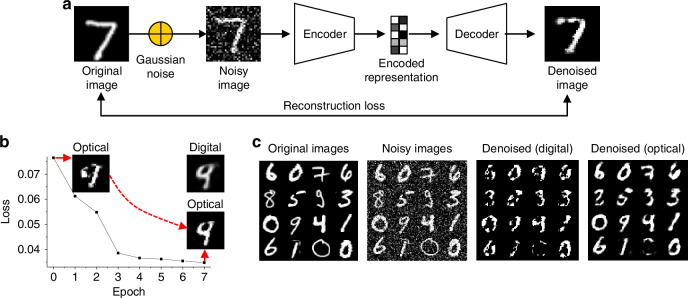
**Application of the optical autoencoder as a DAE**. **a** Schematic of the DAE training process, involving pairs of clean and noisy images to train the model. **b** Loss reduction over training epochs, demonstrating the effectiveness of the DAE in denoising input images. **c** Examples of noisy input and denoised output images, showing the improvement in image quality after denoising

The optical autoencoder network can also be trained as a DAE to remove noise from input data (Fig. 5a). This training process involves providing the DAE with pairs of clean and noisy images, enabling it to learn to minimize the difference between the reconstructed output and the clean images^42^. Gaussian noise, with a standard deviation of *σ* = 0.4, was added to create the noisy inputs, where σ is defined within the normalized range of 0‒1. Under this condition, the PSNR of the noisy images is ~8 dB. Despite the high noise level, the optical DAE demonstrated robust performance, achieving a loss reduction from 0.0765 at epoch 0 to 0.0348 at epoch 7 (Fig. 5b). During iterative optical fine-tuning, the DAE effectively denoised the inputs by focusing on essential features, enhancing the quality of reconstructed images by progressively blackening the background and whitening the core features (Fig. 5c).

However, certain noisy images, such as the digits “8,” “9,” and “0” in Fig. 5c, were not reconstructed correctly. These digits share the common characteristic of having relatively thin structures, which makes it challenging to distinguish their features from the noise. This thinness increases the likelihood of feature distortion or loss during the denoising process, leading to incorrect reconstructions. Additional examples of noisy inputs and reconstructed outputs are presented in Supplementary Fig. S7, illustrating the robustness and limitations of the system under high-noise conditions. These results underscore the versatility and effectiveness of DAEs in improving data quality and extracting valuable information across various fields.

### Implementation of VAE and GAN for MNIST image generation

**Fig. 6:**
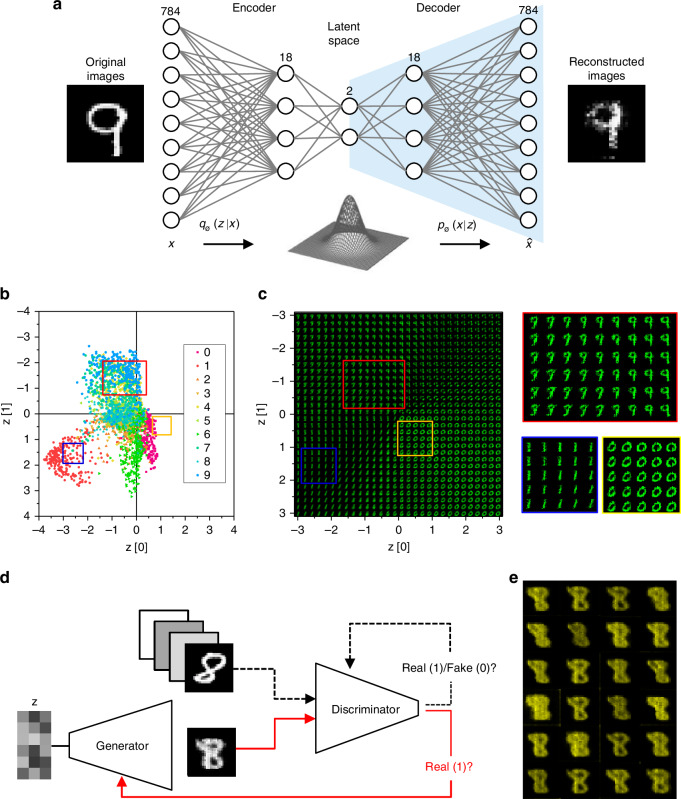
**Implementation and results of a VAE and GAN using type 2 multipliers**. **a** Network structure of the VAE, highlighting the decoder implementation. **b** Visualization of the 2D latent space mapped by the VAE for MNIST digits, showing clustering of similar digits. **c** Reconstructed image array from the 2D latent space (left panel), with enlarged images (right panels) for the regions highlighted in red, blue, and yellow boxes. **d** Schematic of the GAN architecture with the optical multiplier used in the second layer of the generator network. **e** Example of an image generated by the optical GAN

VAEs are designed for generative tasks, differing from traditional autoencoders by employing a probabilistic approach^42^. In a VAE, the encoder maps the input data into a latent space that typically follows a Gaussian distribution, while the decoder reconstructs data from a latent vector sampled from this distribution, generating images similar to the original inputs. Figure 6a illustrates the network structure and operation implemented in our prototype, featuring a two-layer encoder and decoder network. Since the encoder is primarily used during training and the decoder is responsible for generating new images, our ONN-based VAE focused on implementing only the decoder. Specifically, the initial step of decoding, which involves transforming the 2-dimensional latent space into 18 intermediate features, requires minimal computation (2 × 18 MAC operations). However, the subsequent step, which expands these 18 features into 784 output features, involves significantly more computation (18 × 784 MAC operations). Therefore, we experimentally implemented the second layer of the decoder network, while the initial layer was processed using digital computation.

The effectiveness of the VAE is demonstrated by mapping the 10 MNIST digits into a 2-dimensional latent space, as shown in Fig. 6b, with Fig. 6c providing a visualization of the decoded latent space^15,43^. Similar digits tend to cluster together, indicating that the VAE effectively compresses and classifies the data. However, these clusters show inconsistent shapes and significant overlap—a phenomenon that is expected given that certain numbers naturally resemble each other and because our network may not be deep enough (red boxes in Fig. 6b, c). Despite these challenges, the results demonstrate the potential of the VAE for generating diverse digit images.

GANs consist of two neural networks—the generator and the discriminator—that operate in a competitive setup^44^ (Fig. 6d). After training, the generator’s decoder is used to create new images from random latent space vectors. In our prototype, the optical multiplier implements the MVM operation for the second layer of the generator network, a more complex layer (18 input neurons and 784 output neurons). Although the double-layer decoder is relatively shallow, which limits its capability to generate simpler images such as the MNIST digit ‘8’, Fig. 6e exemplifies the potential of optical GANs for generating realistic images from random noise vectors sampled from a standard normal distribution.

## Discussion

Assessing the energy efficiency and speed of ONNs has been extensively explored in the literature, with many studies focusing on inference tasks or encoding processes^6,19,28,34,36,45^. Many ONN prototypes, including our own, consume several to tens of watts from conventional experimental devices such as displays (or lasers), LCDs (or SLMs), cameras, and digital control units^5,34,35,46^. The speed is often bottlenecked by the low operating frequencies of these devices, which typically have a large number of pixels (e.g., frame rates ranging from ~100 to 1000 Hz for SLMs and high-speed cameras)^5^. However, by minimizing optical losses and optimizing pixel usage, ONNs can achieve superior performance. Recent experimental demonstrations have shown that ONN components can achieve extremely low energy per operation and high throughput. For instance, Bernstein et al. demonstrated a proof-of-concept experiment performing 19,600 MAC operations and further projected that, with optimized implementations and wider DNN layers (N = K ≈ 1000), a single-shot ONN system could achieve a theoretical energy consumption of ~10 fJ/MAC and a throughput of ~petaMAC/s^34^. Similarly, Song et al. reported a proof-of-concept optoelectronic network operating at ~11.6 GOPS/W (≈0.086 nJ/MAC), with projections up to ~2.92 TOPS/W ( ≈ 0.5 pJ/MAC) when scaled with larger arrays and faster modulation^47^. Most strikingly, Wang et al. have demonstrated computation in the single-photon regime, using less than one photon per MAC (~10^−3^ aJ/MAC)^36^. These state-of-the-art results apply most directly to the optical encoding stage (Type 1 MVM) of neural networks, but by extension, they also support the feasibility of equally efficient optical decoding stages (Type 2 MVM) when properly optimized.

Given the current limitations of our proof-of-concept system—which operates with OLED displays at ~60 Hz and cameras at 165 FPS—the primary objective has been to validate the fundamental feasibility of our ONN multipliers for encoding and decoding tasks, rather than to demonstrate high-speed or highly energy-efficient performance. In our prototype, the OLED’s maximum emitted intensity was approximately 100 µW/cm², with the actual input averaging around 50 µW/cm² (yielding roughly 200 µW for a 4 cm² area of decoder network). In practical implementations, this OLED display would be replaced with significantly more efficient semiconductor emitters, such as inorganic LED or laser-diode (LD) arrays, which offer far superior wall-plug efficiencies (over 50%) and support high-frequency, nanosecond-scale pulsed operation. Pulsed operation allows the emitter to be active only during inference, dramatically reducing average power consumption compared to continuous operation. On the detection side, employing a compact 28×28 (784-pixel) high-speed CMOS sensor array enables measurement speeds in the MHz range (e.g., 1–10 MHz or higher) using modern high-performance CMOS with ASIC integration. For example, assuming an LED with ~50% wall-plug efficiency, operation at 1 MHz (1 µs/frame) or 10 MHz (100 ns/frame), and accounting for typical optical path losses ( ~ 94%), the optical energy of the decoder is estimated to be in the range of ~0.04–0.4 nJ per inference. Even after including practical electronic overhead (e.g., ADC and readout circuitry), the total per-inference energy remains comfortably below the ~10 nJ benchmark typically cited for high-efficiency electronic DNN accelerators.

**Fig. 7:**
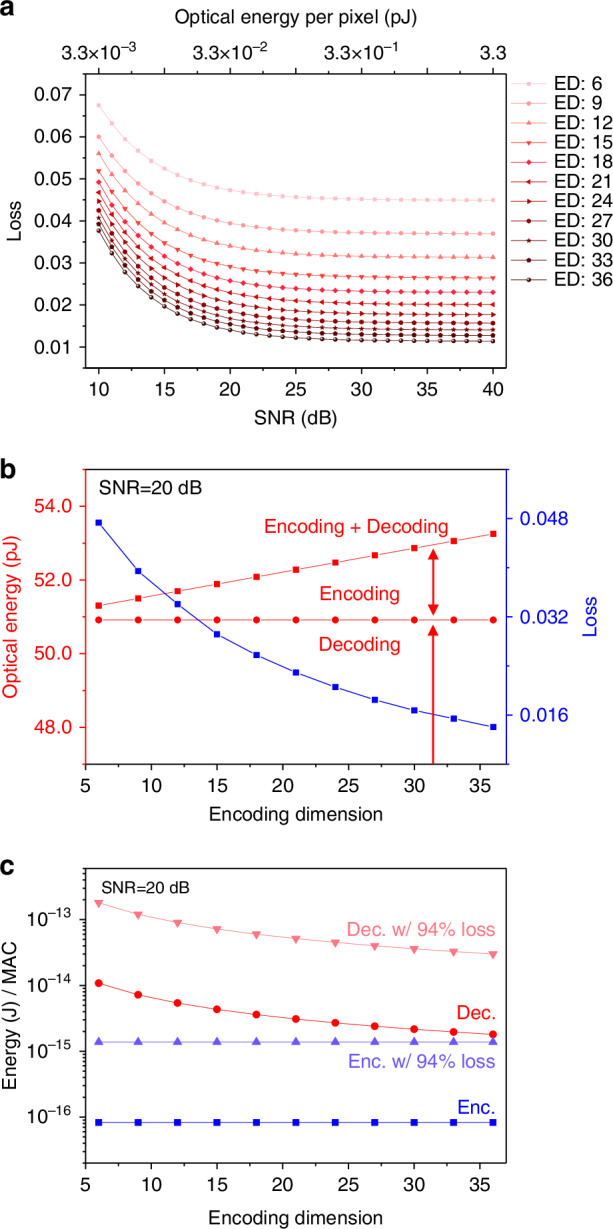
**Analysis of MSE loss and energy efficiency across different SNR levels and EDs in the ONN-based autoencoder**. **a** MSE trends for MNIST test data across varying SNR levels (bottom *x*-axis), with corresponding optical energy per pixel required for achieving MVM operations (top *x*-axis). The systems discussed here are trained with built-in noise tolerance mechanisms, incorporating on-system iterative tuning conducted with noise present, while models without such mechanisms are shown in Supplementary Fig. S8. **b** Total optical energy used for encoding and decoding tasks (red dots and left *y*-axis) and MSE loss, depending on the ED, estimated at an SNR of 20 dB. **c** Estimation of energy per MAC operation for encoding and decoding tasks, with and without considering multiplicative energy losses (94% total, ~6% efficiency) from electrical-to-optical conversion (30%) and transmission losses in components such as LCDs/SLMs (80%) and lenses (1%). This analysis focuses solely on input optical power and does not account for the additive operating power of active devices (e.g., OLEDs and LCDs)

Next, we estimate the energy input required for robust operation, where the process of optical rays reaching the sensors and being converted into signals is critical^48^. For this, we first modeled our system—featuring a Type 1 encoder and a Type 2 decoder—assuming no optical losses and using a photodetector array with 80% quantum efficiency and 1 electron readout noise (see Methods for details). Figure 7a illustrates the trends of MSE loss for MNIST test data across varying SNR levels, along with the corresponding optical energy required for MVM operation per pixel detector. The models presented here are trained with built-in noise tolerance mechanisms, accounting for on-system iterative tuning performed in the presence of noise, while models without such mechanisms are shown in Supplementary Fig. S8. As the SNR and/or ED increase, performance improves. For example, at an SNR of 20 dB (where each pixel detector requires 32.47 fJ of optical energy), increasing the ED from 6 to 36 reduces the loss from 0.0473 to 0.014 (Fig. 7b, blue dots; see Supplementary Fig. S9a for plots at various SNR values).

Additionally, increasing the ED linearly raises the total optical energy required by the encoder, as more detectors are involved, each requiring a certain amount of optical energy for a given SNR (Fig. 7b, red dots). In contrast, the energy required by the decoder remains constant, as it always has a fixed number of detectors (784 in this case), corresponding to the output neurons. While the decoder consumes more overall optical energy due to its larger number of output neurons, the increase in energy consumption with higher ED is minimal (Fig. 7b, red dots; see Supplementary Fig. S9b for plots at various SNR values). On the other hand, increasing ED comparatively improves performance by reducing loss. While increasing SNR also decreases loss, its effect becomes negligible at SNR above 30 dB, as the energy requirement rises exponentially with little improvement in loss (Supplementary Fig. S9c). This analysis of the relationship between performance, energy, and ED can be valuable for designing energy-efficient systems that balance cost and performance.

The Type 2 decoder scheme demonstrated in this work is particularly well-suited for scenarios where the output dimensions exceed the input dimensions. For practical applications requiring larger encoding dimensions (*K* > 18), several scalability strategies can be employed. First, the scalar multiplication mechanism can be extended by increasing the number of input pixels and overlapping regions, allowing the system to handle larger output dimensions without significantly increasing optical complexity. Second, customized lens and/or mirror arrays with precise magnification control and alignment can ensure accurate scalar multiplication and image overlap (see “Materials and Methods” in Supplementary Information for details). Additionally, high-resolution LCDs or increased LCD segments, combined with multi-channel light modulation techniques such as RGB multiplexing, enable efficient weight storage for larger K. Third, parallelization using multi-beam optical paths can further enhance scalability by enabling simultaneous processing of larger blocks, addressing latency challenges while maintaining efficiency. Finally, despite the increased total optical energy requirements, the energy per MAC operation remains low due to the inherent parallelism of optical processing. Optimized light sources, such as LEDs or laser diodes, and reduced optical losses can ensure competitive or even improved energy efficiency, as shown in Fig. 7c. These strategies collectively highlight the scalability potential of the Type 2 decoder scheme for practical and complex applications.

Although the decoder requires more optical energy per MVM operation due to its larger number of output neurons, the energy per MAC remains low, around 10^−2^ to 2 × 10^−3^ pJ (corresponding to 200–1000 TOPS/W) (Fig. 7c, red circles). However, in-field implementations must account for energy losses during electrical-to-optical conversions and while traversing free space. Assuming a 94% energy loss (see Methods), the energy per MAC for encoding and decoding is estimated at 1.38 fJ and 0.18–0.03 pJ (Fig. 7c, red triangles), respectively, which remains competitive with state-of-the-art GPUs/TPUs (~0.1‒10 pJ/MAC)^36^.

It is noted that this energy estimate excludes the minor energy required for final subtraction operations, which constitute only a small fraction of the total MVM energy (0.01‒0.1 pJ per subtraction operation in 8-bit digital systems^49^. Additionally, the energy needed for hardware operation—such as driving optical components, data conversion, and signal transmission—must be considered, as these factors can increase overall energy consumption. Although minimal compared to core MVM operations, these aspects should be considered for a complete assessment of the system’s energy efficiency in practical applications. An innovative approach for optical subtraction can be achieved, for instance, through the use of destructive interference by precisely controlling the phase of coherent light beams^50^. Integrating this approach with optical nonlinear activation functions could enable fully optical implementations of advanced computational architectures^5,6,24^. By eliminating back-end digital processing, such strategies could offer near-instantaneous processing speeds, vastly improved energy efficiency, and the ability to handle massive parallel processing for high-resolution image reconstruction.

## Materials and methods

### ONN hardware for autoencoder

The hardware setup for our ONN-based autoencoder, optimized for optical matrix-vector multiplication (MVM) in both image encoding and decoding, included key optoelectronic components: OLED displays (TFX156T, Hansung Co., Ltd, Korea) for generating fan-out input images, LCD panels (708H, Viva Science Technology Co., Ltd, China) for modulating weight matrices, and scientific cameras (CS135MU, Thorlabs, Inc.) for capturing output beam intensities. These components were complemented by optical elements such as mirrors, lenses, and beam splitters for focusing and overlapping images (Fig. 3a).

For the encoder network, we implemented a Type 1 MVM using green OLED pixels to represent input images, which were modulated by the LCD panel to perform pixel-wise scalar multiplication. The 28×28 pixel input images were fan-out replicated across the OLED display, corresponding to the weight matrix patterns displayed on the LCD. After modulation, the images were optically demagnified using lenses and detected by the scientific camera, performing simultaneous MVM operations.

For the decoder network, a Type 2 MVM was implemented using all RGB channels of the OLED and LCD panels, increasing throughput and improving alignment in the fan-in process. Positive and negative weight elements were separated into distinct OLED blocks, and each input pixel was magnified and multiplied by the weight matrix on the LCD. The modulated images were overlapped using mirrors and beam splitters, generating a 784-pixel output image (Supplementary Fig. S4). For larger encoding dimensions (e.g., ED of 18), multiple images were captured by the camera and digitally combined. Alternatively, this could be done via analog methods using pixel binning or summing currents in photodetector arrays.

### Training and operation of autoencoder networks

A digital version of the autoencoder was first trained using Python, NumPy, and TensorFlow, with ReLU as the encoder activation function and sigmoid for the decoder (for multi-layer decoders, ReLU was used in the first layer and sigmoid in the last layer). The model was trained on MNIST, Fashion-MNIST, and K-MNIST datasets, using MSE as the loss function and gradient descent for optimization, continuing for approximately 100 epochs. The trained weights were then exported for use in the ONN hardware.

The ONN-based autoencoder operated similarly to the digital version, with LabVIEW-based software controlling the OLED displays, LCD panels, and cameras. During operation, variations in RGB intensity ratios (R:G:B = 1:0.9:0.6) and a ~ 30% intensity reduction through mirrors were observed. Additionally, non-linear light transmission through the LCD pixels caused further deviations. To mitigate these issues, corrections were applied to the trained weights, and on-system iterative tuning with backpropagation was employed, improving reconstruction accuracy using 3000 training samples and 500 test images for validation.

### Implementation of VAE and GAN for MNIST image generation

We implemented a VAE with a two-layer encoder and decoder to generate MNIST images. The decoder reconstructed images from a 2D latent space, focusing on the 784-output dimension using optical MVM operations. Similarly, a GAN was implemented, with the generator trained to create images from random latent vectors, focusing on the digit “8”. The generator’s second layer (18 input to 784 output neurons) was implemented optically using Type 2 MVM.

### Simulation of optical MVM strategies

Simulations were conducted to compare the performance of Type 1 and Type 2 MVM strategies in the decoder. The autoencoder, trained to reconstruct 28 × 28 pixel MNIST images, was simulated with 784 input/output neurons and 16 encoding neurons. Weights were split into separate matrices for positive and negative values. For Type 1, fan-out replication and downscaling of input images were simulated. In contrast, Type 2 simulated magnified input pixels and scalar multiplication with the weight matrix, followed by overlapping the resulting images to perform element-wise summation. Performance was measured using MSE across 3000 MNIST test images.

We also evaluated robustness by introducing image distortions (0–0.4% shearing and misalignment) and assessing their impact on MSE. Type 1 distortions were applied before multiplication, while Type 2 distortions were applied during pixel summation.

### Predicting energy efficiency in autoencoder architectures

We modeled the energy efficiency of optical MVM operations for both encoding and decoding tasks using optimized optical sources and detectors. The system used a photodetector array with 80% quantum efficiency and 1 electron readout noise. The performance was evaluated under varying SNR levels (10–40 dB) and encoding dimensions (EDs between 6 and 36). Energy consumption was calculated based on ED and SNR, which ultimately determined the MSE values and the optical energy required per pixel detector during MVM operations.

Initial simulations assumed ideal conditions without optical losses. However, considering real-world implementations, we included an additional energy loss (up to 94%) to account for factors such as electrical-to-optical conversion efficiency (30%) in optical input signal generation (e.g., OLED, LED displays, LED/LD arrays) and losses in optical components like LCDs, SLMs, and lenses (for further details, refer to “Discussion” in the Supplementary Information).

## Supplementary information

Supplementary Information for Image processing with Optical matrix vector multipliers implemented for encoding and decoding tasks

## Data Availability

All data are available in the main text or the supplementary information. Additional data related to this paper may be requested from the corresponding authors upon request.
