# Supplementary material for: Image processing with Optical matrix vector multipliers implemented for encoding and decoding tasks
Source: Light Sci Appl. 2025 Jul 22;14:248. doi: 10.1038/s41377-025-01904-z (PMC12284195; doi:10.1038/s41377-025-01904-z)
Supplement: Supplementary file 1 — Supplementary Information for Image processing with Optical matrix vector multipliers implemented for encoding and decoding tasks [file 41377_2025_1904_MOESM1_ESM.pdf]

Supplementary Information for Image processing with Optical matrix vector multipliers implemented for encoding and decoding tasks

Minjoo Kim[a†], Yelim Kim [a†], and Won Il Park[a]*

[a]Division of Materials Science and Engineering, Hanyang University, Seoul, 04763, Republic of Korea

[†]*These authors contributed equally to this work*

*wipark@hanyang.ac.kr

KEYWORDS: Optical Neural Networks (ONNs), Optical Matrix-Vector Multiplier, Autoencoder, Optical Decoding, Scalar Multiplication, Generative Models

1. Experimental Setup

1.1. ONN Hardware for Autoencoder

The hardware setup for the Autoencoder, specifically designed for optical MVM in image encoding and decoding, included key optoelectronic components: OLED displays for generating fan-out input images, LCD panels for representing the weight matrix, scientific cameras for detecting output beam intensities, and additional optical elements (e.g., mirror, lens, beam splitter) for image focusing and overlapping (Fig. 3a). The 15.6-inch OLED displays (TFX156T, Hansung Co., Ltd., Korea) and 7-inch LCD panels (708H, Viva Science Technology Co., Ltd., China) feature arrays of approximately 4 million (3840×2160) and 2 million pixels (1920×1080), respectively. Each pixel on both the OLED and LCD is divided into sub-pixels for red, green, and blue colors, with a refresh rate of 60 Hz.

1.2. Optical Matrix-Vector Multiplier for Encoder Network

For the encoder, we implemented a Type 1 optical MVM using green pixels on the OLED display to represent input image patterns. The LCD panel, affixed to the OLED screen, dynamically modulated light transmission for pixel-wise scalar multiplication. For optical fan-out, the input image (28×28 = 784 pixels) was replicated across the OLED screen into arrays corresponding to the weight vectors displayed on the LCD panel. This maintained a one-to-one correspondence between each fan-out input image element and each weight matrix element. For example, with an ED of 6 and weights clamped to positive values, the fan-out input image blocks and weight pattern blocks (each 28×28 pixels) were displayed in 6 arrays (Fig. 3a, encoder). In cases with an ED of 18 and weights including both positive and negative values (Fig. 4), 36 arrays (2×18) were used, with all blocks arranged in a 6×6 configuration.

After modulation through the LCD panel, the fan-out input images were demagnified using optical lenses, and their intensities were measured by a scientific camera (CS135MU, Thorlabs,

Inc.), enabling simultaneous MVM operations. Given the fine pixels of the camera, focusing individual feature maps onto each pixel is challenging, necessitating the summation of light signals across pixel arrays. Alternatively, these operations can be done in an analog fashion by binning camera pixels or using a larger photodetector array.

1.3. Optical Matrix-Vector Multiplier for Decoder Network

We implemented a Type 2 optical MVM for the decoder. Unlike the single-color encoder, the decoder utilized all RGB color channels in both the OLED display and LCD. This approach not only increased the operation throughput per unit area but also improved image alignment during the fan-in process, reducing performance degradation caused by misalignment.

In the decoder network, using only positive weights led to a notable performance drop compared to utilizing both positive and negative weights. For instance, in the case shown in Supplementary Fig. S2 (ED of 6), clamping the weights to positive values during digital training led to an MSE of 0.23, whereas using both positive and negative weights reduced the MSE to 0.044. Additionally, when clamping only the encoder weights, despite an initial performance drop during training, nearly identical MSE loss values were achieved after the training process.

The Type 2 MVM operation was implemented in two ways. First, as depicted in Fig. 3, encoded data with a dimension of 6 was represented using the RGB pixels of two OLED displays. The Type 2 MVM operation required 4 blocks of 28×28 pixel images to represent the weights, with the upper and lower blocks corresponding to the positive and negative elements of each row vector in the weight matrix (Fig. 3c), respectively. For the scalar multiplication operation, each pixel on the OLED display was magnified and duplicated to correspond with the weight blocks on the LCD.

Within each block, color-wise scalar multiplication was performed as the encoded inputs passed through the modulating LCD weights. The resulting images were then overlapped using mirrors and a beam splitter, producing a pair of 784-pixel images captured by a scientific camera (CS135MU, Thorlabs, Inc.) equipped with optical lenses. Digital computations, including subtraction and sigmoid activation, were then performed to complete the decoding process and generate the final reconstructed images.

For EDs larger than 6 (e.g., Fig. 4), instead of merging multiple images using mirrors and beam splitters (or a custom-designed x-cube), we captured multiple images with a camera and combined them digitally. In principle, these digital operations could be conducted in an analog manner by binning camera pixels or electrically connecting specific pixels across images to sum photodetector output currents according to Kirchhoff's current law[1,2]. With the use of coherent, single-wavelength light sources like laser beams, optical subtraction can also be achieved through destructive interference by precisely controlling the phase of the overlapping beams[3-5].

1.4. Training of Autoencoder Networks

We first implemented a digital twin of the autoencoder network using Python, with NumPy and TensorFlow. This served as a preliminary step to obtain weight parameters for future implementation in ONNs. The encoder utilized ReLU as the activation function, while the decoder used sigmoid. The digital autoencoder network was trained on 60,000 training images from the MNIST, Fashion-MNIST, and K-MNIST datasets until the MSE values reached a minimum level, typically after around 100 epochs with a batch size of 200. For the scenario depicted in Fig. 3, training and testing were conducted using datasets that included only the 0, 1, and 2 classes from the MNIST dataset, while for all other cases, the dataset comprising all

10 classes was used for both training and validation. Weights were initialized randomly and updated using gradient descent with MSE as the loss function.

Weights were initialized using different methods depending on the constraints of the model. For cases where both positive and negative weights were required, the Glorot Uniform Initialization (Xavier Initialization) method[6] was used. Weights were sampled from a uniform distribution within the range:

$$W \sim Uniform\left[-\sqrt{\frac{6}{fan\_in + fan\_out}}, \sqrt{\frac{6}{fan\_in + fan\_out}}\right]$$

where *fan_in* and *fan_out* represent the number of input and output neurons in the layer, respectively. This approach improves training stability and convergence speed.

For models requiring only positive weights, weights were initialized using a random uniform distribution within the range of 0–1. This ensured that all weights started as positive values, aligning with the model's design constraints. Additionally, during ONN training, negative weights in the weight matrix were clamped to zero after each parameter update to maintain positivity.

The trained weights for both the encoder and decoder were saved to CSV files for further use in ONN-based autoencoder implementations.

1.5. Operation of ONN-based Autoencoders

Our ONN-based autoencoder network, designed to operate similarly to digitally implemented autoencoder networks, was implemented using LabVIEW-based code. This code controlled the OLED displays, LCD panels, and cameras to produce results from optically performed MVM operations, as described earlier. It also handled forward propagation for image encoding and decoding, as well as backward propagation for training and weight updates.

As described in previous section, the digital twin of the autoencoder network was pre-trained to obtain initial weight parameters, which were subsequently imported into the ONN-based autoencoder network implemented on the hardware system. We observed that the intensity of the measured images from the camera varied across RGB colors, with R:G:B intensity ratios of 1:0.9:0.6. Additionally, images passing through mirrors (left blocks) showed approximately a 30% reduction in intensity compared to those that did not. The light transmission through each pixel of the LCD also varied non-linearly with the weight values represented by the R, G, and B color indices. Despite applying corrections to the trained weights to minimize these optical errors, performance degradation was still observed during the evaluation of our ONN using digitally pre-trained parameters, specifically an increase in reconstruction loss (MSE).

To address this, we implemented on-system iterative tuning by incorporating an error-backpropagation algorithm into the LabVIEW code[6]. This tuning process used a minibatch size of 100 and was performed over 30 iterations per epoch, processing a total of 3,000 training images (MNIST, Fashion-MNIST, and K-MNIST), with 500 images from the test datasets employed for validation. During this process, reconstructed images were compared to their original counterparts to calculate the reconstruction loss (MSE). This loss was then used to iteratively adjust the weights of the network, correcting for issues such as alignment errors, lens distortions, and non-linear light transmission. This iterative tuning directly contributed to the improvements in reconstruction accuracy observed in our results, aligning the system's performance closer to that of digital benchmarks.

After iterative tuning (at epoch 5 for MNIST and epoch 6 for Fashion-MNIST and K-MNIST), we evaluated our ONN's generalization capability using 3,000 test images, including the 500 validation images utilized during training. The evaluation metrics included MSE, PSNR, and cosine distance, along with classification performance based on the reconstructed

images. These comprehensive assessments demonstrated the robustness of our ONN in achieving reconstruction quality comparable to digital implementations.

The denoising autoencoder was implemented in a similar manner, except that noise was added to the input data during training (however, MSE was calculated between the original, pre-noise image and the reconstructed image).

1.6. Implementation of VAE for MNIST Image Generation

For the VAE, satisfactory performance was not achieved with a single-layer encoder and decoder, so both were expanded to two layers (Fig. 5a). ReLU was used as the activation function in most network layers, except for the last layer of the decoder, which used a sigmoid function. During training, the encoder compressed the 784-dimensional input into 18 intermediate features, and then into a 2-dimensional latent space, providing the mean and variance for a Gaussian distribution. The decoder reconstructed the data by expanding this 2-dimensional latent space back into 18 intermediate features, and finally into 784 output features, generating images based on the latent vector sampled from the Gaussian distribution.

The main function of a VAE is to generate new images by sampling from the latent space, and in practical applications, only the decoder is typically used. Therefore, in our ONN-based VAE, we focused on implementing only the decoder. Specifically, the first step of decoding from the 2-dimensional latent space to 18 intermediate features requires minimal computation (2×18 MAC operations), while the next step, expanding from 18 intermediate features to 784 output features, involves significantly more computation (18×784 MAC operations). We therefore experimentally implemented the second stage MVM of the decoder network optically.

1.7. Implementation of GAN

A GAN was implemented to generate MNIST images, focusing on the digit '8'. The GAN consisted of a generator and a discriminator, both structured as sequential neural networks. The generator network accepted an 18-dimensional latent space vector as input, passing it through two fully connected layers: the first with 18 units using ReLU activation, and the second with 784 units (corresponding to a 28×28 pixel image) using sigmoid activation to produce the generated image. The discriminator was designed to distinguish between real and generated images, consisting of two fully connected layers: the first with 128 units using ReLU activation, and the second with a single unit using sigmoid activation to output the probability of the input image being real.

Similar to the VAE, the GAN generates new images from random latent space vectors. Therefore, after training, only the generator's decoder is used to create new images. In this experiment, the MVM operation of the second, more complex layer of the generator (with 18 input neurons and 784 output neurons) was implemented optically using the Type 2 MVM approach. The MNIST dataset, limited to the digit '8', was used to construct the training and testing sets. Following digital training to optimize the weights of both the generator and discriminator, the trained generator's network weights were imported for optical implementation. The input to the generator was a random noise vector sampled from a standard normal distribution (mean of 0 and standard deviation of 1).

2. Simulation Method for Evaluating Optical MVM Strategies

To assess the effectiveness of different optical MVM strategies in decoding, a detailed simulation was conducted using an autoencoder model designed to reconstruct 28×28 pixel images of handwritten digits from the MNIST dataset. The autoencoder consisted of 784 input/output neurons and 16 encoding neurons, with fully connected layers comprising both the encoder and decoder networks. The encoder compressed the input data into a 16-dimensional latent space, while the decoder reconstructed the original image from this compressed representation.

The autoencoder was trained using the MSE as the loss function, with gradient descent optimization. Training continued until the MSE values stabilized at a satisfactory minimum level. Once trained, the weights were transferred to a simulation environment to compare the performance of Type 1 and Type 2 MVM operations.

In the simulation, the decoder's weight matrices were split into two components to separately handle positive and negative elements: one matrix representing positive weights and the other representing negative weights. For the Type 1 MVM operation, the simulation involved a fan-out process applied to the input image, followed by element-wise multiplication with the weight matrix. The resulting images were then processed through a fan-in operation, where the 16×784 pixel images were downscaled to 784 pixels by summing the 4×4 pixel blocks to produce a single pixel value for each of the 784 output vectors. This downscaling process, typically achieved by demagnifying the 16×784 pixel images to 784 pixels during image capture, was simulated by resizing the input image to the specified new size using bicubic interpolation. In contrast, the Type 2 MVM operation was simulated by magnifying the input pixels and performing scalar multiplications with the weight matrix. The resultant images were then overlapped to achieve element-wise summation.

The performance of both MVM methods was evaluated by reconstructing 2D images from the compressed data and comparing the reconstructed images to the original ones using MSE as the evaluation metric. The simulations were specifically performed using 3,000 images from the MNIST test dataset. The MSE values obtained from the Type 1 and Type 2 decoding methods were recorded to assess the accuracy of the reconstructions.

Additionally, the simulation examined the impact of potential image distortions, such as shearing and misalignment (horizontal and vertical shifts), on the MVM performance. A 0–0.4% variation in image shearing and alignment was introduced to simulate these distortions, and their effects on the MSE were evaluated for both MVM methods. For Type 1, the distortions were applied before multiplication with the weight matrix, while for Type 2, they were applied during the summation of the 4×4 pixel blocks after the MVM operation.

3. Predicting Energy Efficiency in Autoencoder Architectures

To evaluate the energy efficiency and performance of optical matrix-vector multipliers (MVMs) used in both encoding and decoding tasks, we conducted simulations and energy estimations considering a system equipped with optical sources and detectors optimized for our system. The goal was to assess the required energy input for robust operation and its impact on overall system performance, particularly considering hardware limitations during the optical signal capture and conversion processes.

We modeled an ONN system with a Type 1 encoder and a Type 2 decoder, assuming no optical losses during the initial simulation. The system featured a photodetector array with 80% quantum efficiency and a readout noise of 1 electron, used for converting optical signals into electrical signals. This setup was applied to evaluate the encoder and decoder's performance under various SNR levels and EDs. The system was trained using the MNIST dataset with an autoencoder model designed to tolerate noise, simulating real-world optical noise during MVM operations[7,8]. Specifically, Gaussian noise corresponding to an SNR of 20 dB was introduced during training to enhance the system's natural noise tolerance.

To measure energy efficiency, the optical energy required per pixel detector during MVM operations was calculated. The total energy consumption was computed based on different ED and SNR levels, ranging from 10 dB to 40 dB. The system's performance was evaluated by calculating the MSE loss across 3,000 MNIST test images. For varying SNR levels, the system was tested at 10 dB intervals, from 10 dB to 40 dB, to observe the effect of noise on MSE values. Additionally, the ED was varied between 6 and 36 to assess its impact on both MSE and overall energy consumption, providing insight into how EDs affect system performance.

The experimental results, as illustrated in Fig. 7 and Supplementary Fig. S9, demonstrate the relationship between SNR, ED, and the optical energy required for MVM operations. Energy efficiency was determined by calculating the energy per MAC operation for both

encoding and decoding tasks. Initial simulations assumed ideal conditions without considering optical losses. However, real-world implementations introduced additional energy losses during electrical-to-optical conversion and free-space propagation. To account for these factors, we modeled a 94% cumulative energy loss, which corresponds to an overall efficiency of approximately 6%. This includes a 30% efficiency for electrical-to-optical conversion of the light source, 80% loss in liquid crystals of LCDs or SLMs, and 1% loss in lenses due to transmissivity, absorption, and scattering effects (i.e., $0.3 \times 0.2 \times 0.99 = {\sim}0.06$). Beam splitters and mirrors, commonly used in optical systems, were not considered in this simulation (despite their straightforward implementation as in the case of Fig. 3a) due to their significant optical losses. A non-polarizing beam splitter divides the incoming light equally, transmitting 50% and reflecting 50%, which introduces a 50% loss per pass. For systems combining multiple (N) images, this results in an exponential decrease in total transmission efficiency $T = (0.5)^N$. While beam splitters and mirrors were employed in our proof-of-concept experiment to achieve image overlap, they are not suitable for practical implementations with high EDs. Instead, energy-efficient alternatives can be realized by optimizing the arrangement of mirrors or lenses to focus and overlap optical beams without light division, minimizing energy losses.

4. Scalability Strategy for Larger Encoding Dimension (ED or K>18) of the Type 2 Decoder

To scale the Type 2 MVM to larger ED (or K), our approach utilizes customized optical elements—such as projection lenses and mirror arrays—that offer finer magnification control and precise alignment. These elements are designed not only to ensure accurate scalar multiplication and image overlap but also to minimize the energy loss typically associated with beam splitters.

Accurate scalar multiplication and proper image overlap are critical for maintaining system performance at high K values. To this end, we incorporate (i) customized projection lenses—high-quality lenses with adjustable magnification and minimal aberrations to ensure consistent scalar multiplication across the entire image field—and (ii) mirror arrays—precisely engineered mirrors with fine adjustment capabilities to control the incident angles and optical path lengths, thereby ensuring that multiple sub-images overlap accurately on the projection screen.

To evaluate the scalability and physical feasibility of our approach for large K, we conducted a preliminary experiment aimed at preventing energy loss from beam splitters. In this experiment, we first verified the physical feasibility of overlapping images using three mirrors when setting the K to 9 (see Fig. S10a, b).

Specifically, an 80×80 pixel lion image was used as the test subject. Each pixel of the image was split into the sum of arbitrary sub-pixel values to generate 9 sub-split images (Fig. S10c). These 9 sub-split images were then grouped into three sets and encoded into the RGB color channels so that each resulting RGB image contains three sub-images. Three such RGB images were projected from different positions using beam projectors. In each image, the sub-images are independently encoded in the R, G, and B channels. The projected images are then incident

on mirrors and reflected onto a screen, where they overlap to form a single composite image. This overlapped image is captured by a monochrome camera, resulting in an 80×80 pixel image.

To further evaluate the results, we generated sub-split images for split counts ranging from 2 to 8 using the same method, overlapped them, and captured the resulting composite images under identical conditions (Fig. S11a). The overlapped images were found to be similar to the unsplit image. Quantitative evaluation using normalized metrics, such as the Structural Similarity Index (SSIM) and Cosine Distance (Fig. S11b, c), further confirmed that the mirror-based overlapping approach maintains low optical loss while providing results that are sufficiently close to the original image. It is also noted that, although the similarity to the original image gradually decreases as the number of overlapped images increases—due to factors such as image misalignment, optical aberrations, nonlinearity in color encoding, and pixel crosstalk or interference—these issues are amenable to further optimization.

These findings indicate that by appropriately utilizing the RGB channels, mirror arrays, and projection lenses, the system can scale efficiently even as the ED increases. For example, by adding two additional lenses each above and below the current setup and using seven mirrors, an ED of 21 is readily achievable. Additionally, since the mirror-based overlapping approach avoids splitting loss, optical loss is minimized. This result strongly suggests that the proposed method will be effective even for larger encoding dimensions.

5. Supplementary Figures and Tables

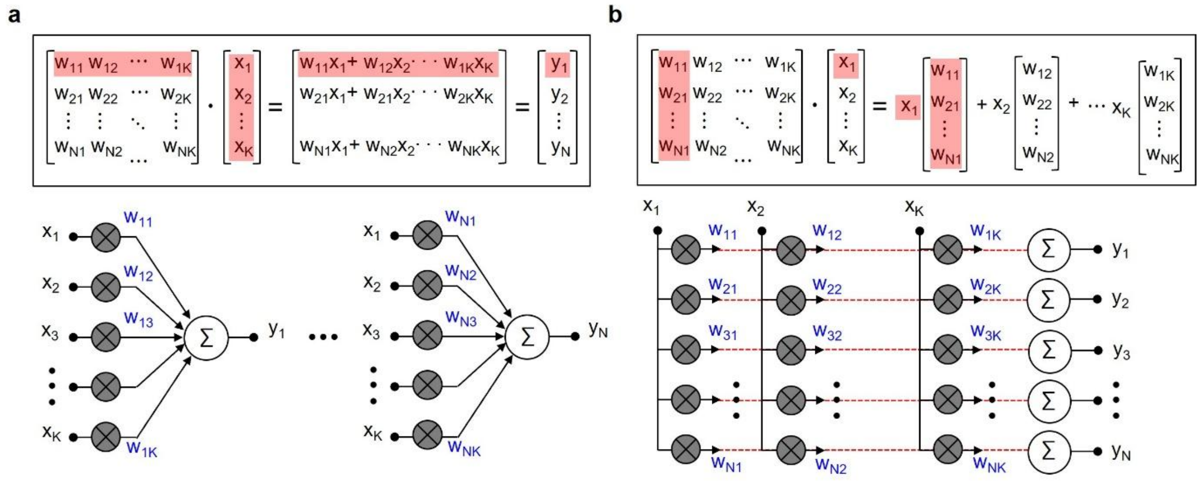

Fig. S1 Mathematical representation of MVM operations and schematic of the procedures for performing these operations. a Type 1 MVM operation involving parallel vector-vector dot products between the input vector with *K* elements and the row vectors with *K* elements in the weight matrix. The red-shaded components highlight the first vector-vector dot product, corresponding to the red lines in Fig. 1b (encoder) and Fig. 1c. **b** Type 2 MVM operation involving parallel scalar multiplications between input elements and column vectors with N elements in the weight matrix. The red-shaded components represent the first scalar multiplication, corresponding to the red lines in Fig. 1b (decoder) and Fig. 1d.

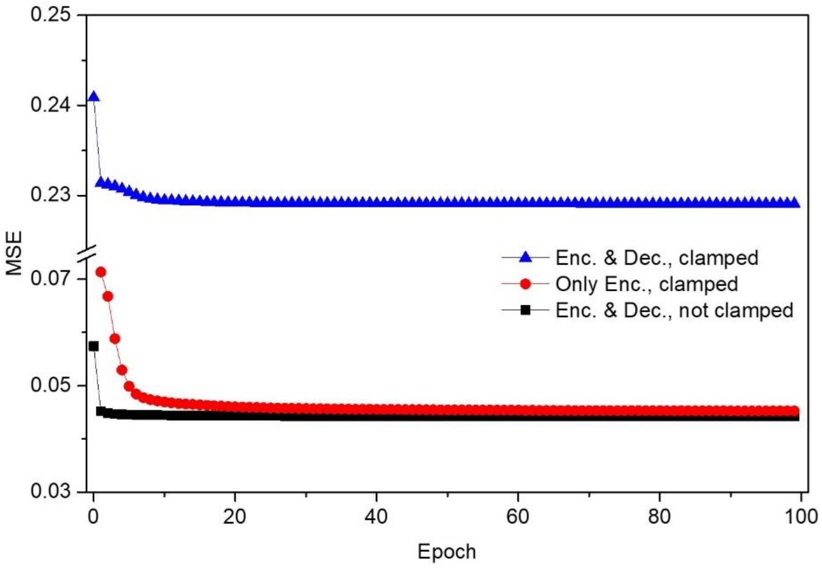

Fig. S2 Performance (MSE loss) comparison of autoencoders with an encoding dimension of 6, showing the effects of weight clamping in the encoder and decoder networks. Black squares and line: both encoder and decoder networks utilize positive and negative weights; initialized using the Glorot Uniform Initialization method[6]; Red circles and line: only the encoder network is clamped to positive weights; initialized using a Random Uniform Initializer with a range of 0–1; Blue triangles and line: both encoder and decoder networks are clamped to positive weights. Clamping the encoder weights to positive values caused minimal performance degradation during training, achieving near-identical MSE loss values after sufficient iterations. However, applying the same constraint to the decoder network led to a significant drop in performance, increasing the MSE from 0.044 to 0.23, demonstrating the critical role of weight flexibility in the decoder network for maintaining reconstruction accuracy.

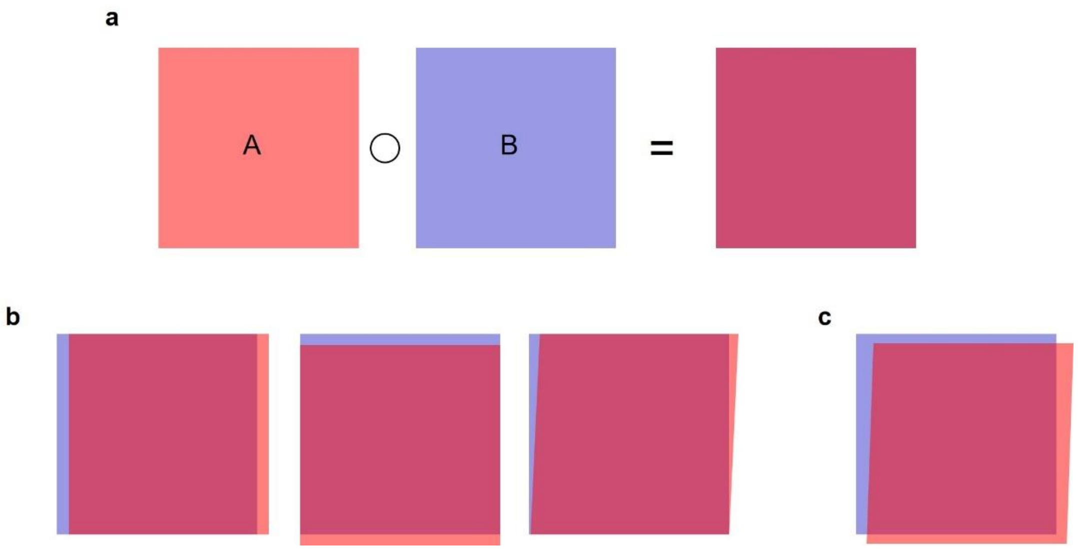

Fig. S3 Optical distortion. a Perfect image overlapping between images A and B (optical distortion = 0%). **b** Image overlapping with either lateral (left) or vertical (middle) misalignment, or shearing (right). **c** Combined distortions, including both misalignments and shearing, considered in our simulation for Fig. 2e. For Type 1 multipliers, misalignment and shearing are applied to the fan-out input patterns in the OLED relative to the weight matrix patterns in the LCD during element-wise multiplications. In Type 2 multipliers, misalignment and shearing occur in the output images after scalar multiplication. These distorted output images are then overlapped and combined into a single image through element-wise summation.

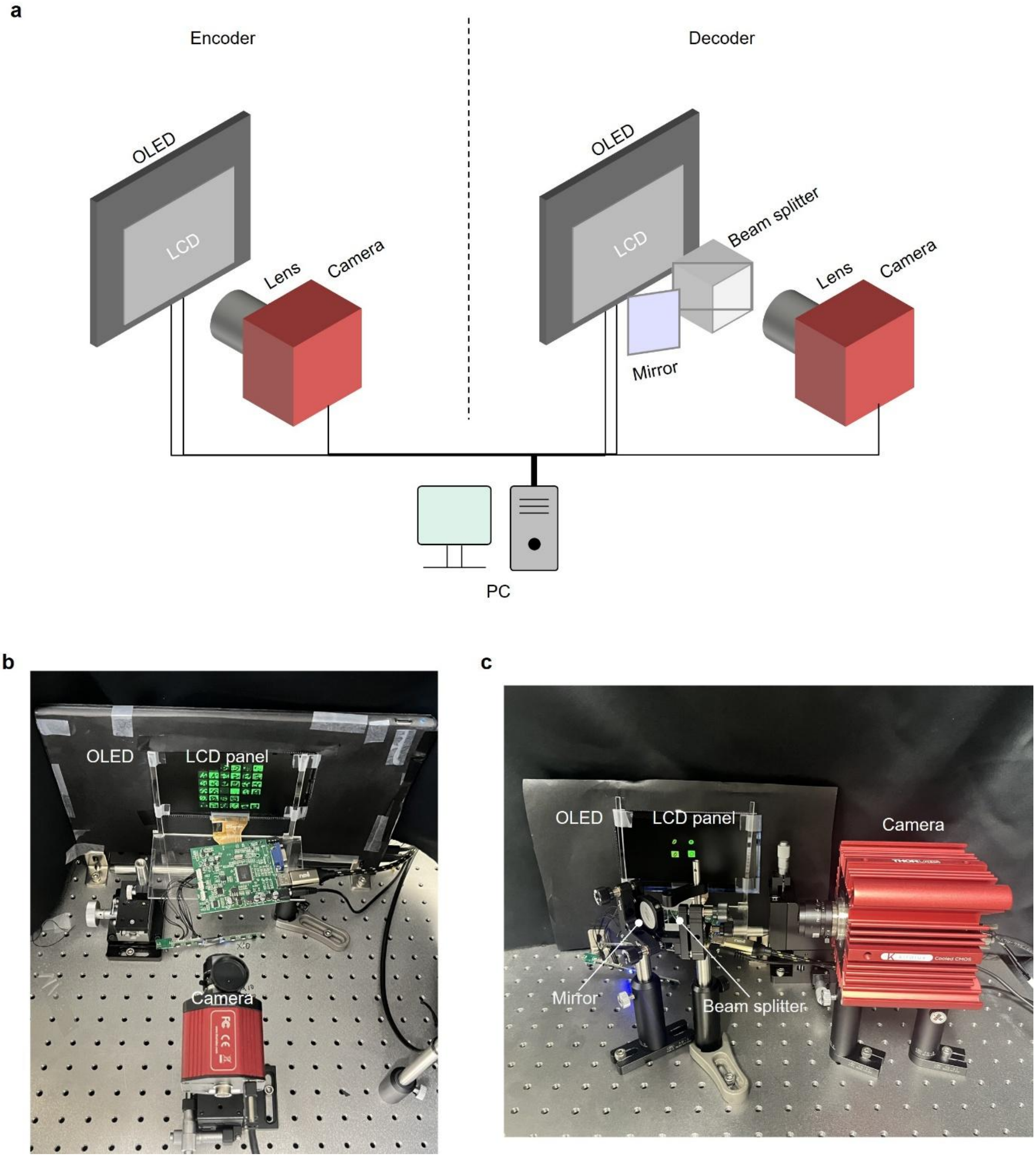

Fig. S4 Hardware implementation. a Schematic of the optical setup. **b, c** Photos of the optical setup used for encoder (b) and decoder (c) corresponding to the schematic shown in (a).

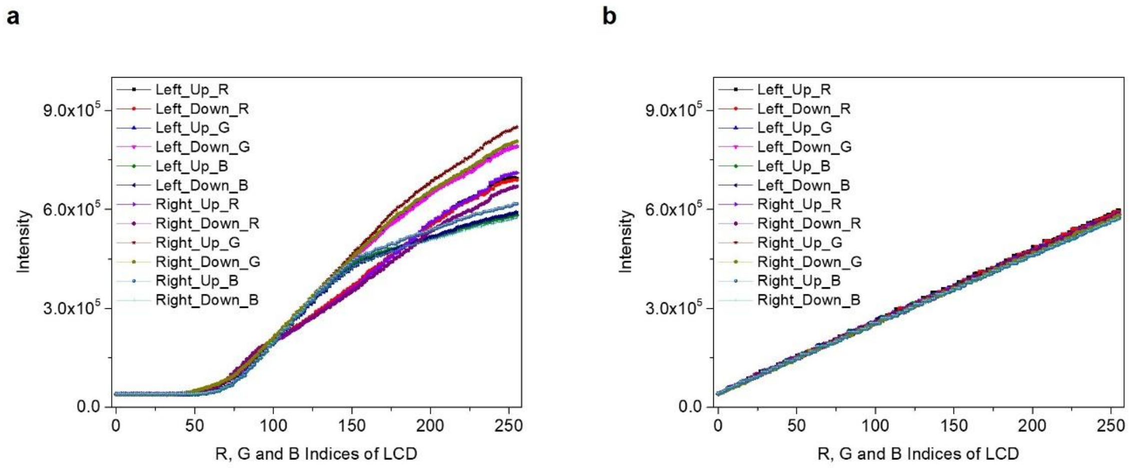

Fig. S5 LCD calibration. a LCD index versus transmitted light intensity expressed in arbitrary units (a.u.) before applying linear and intensity corrections, illustrating the non-linear behavior of light transmittance through the LCD pixels. **b** LCD index response after applying linear and intensity corrections, showing improved linearity and uniform light transmittance.

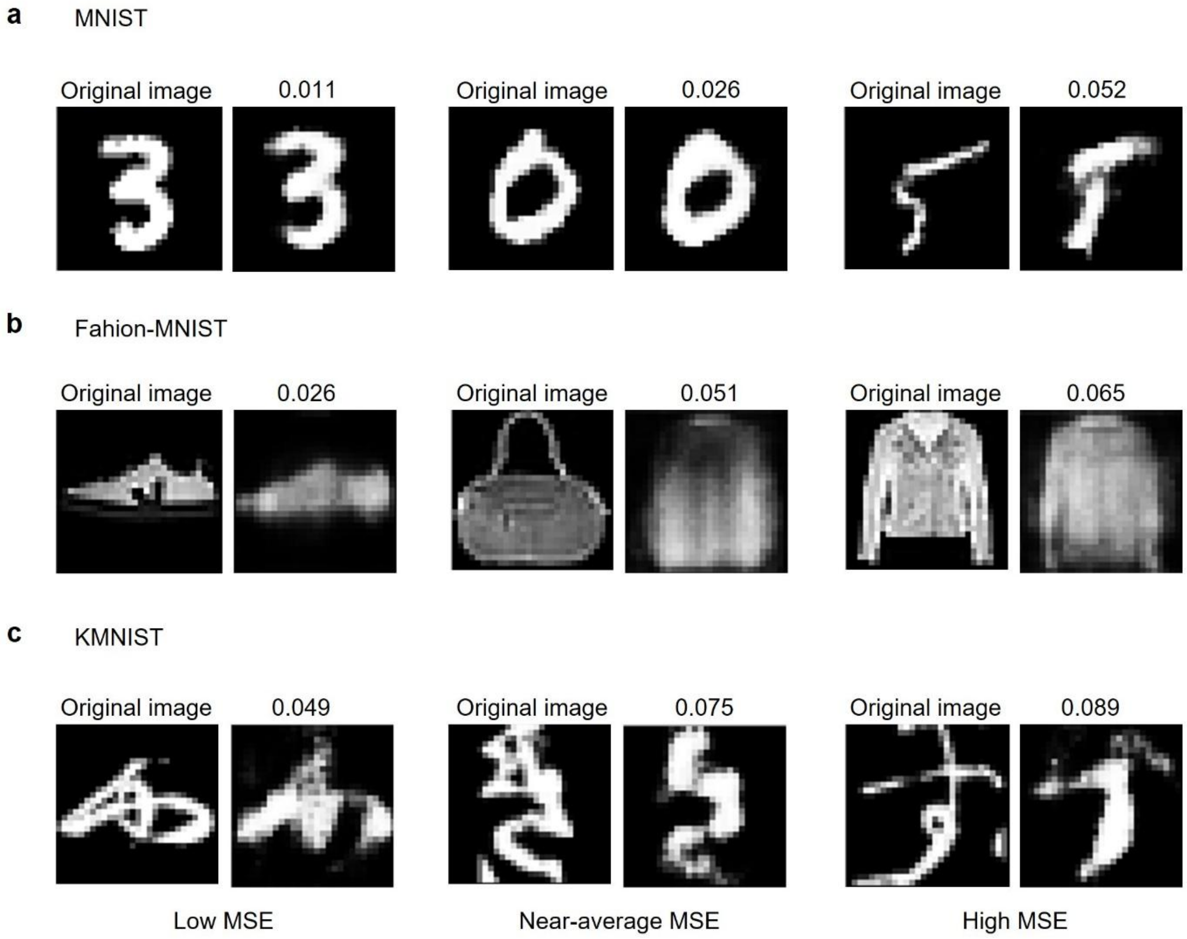

Fig. S6 Representative examples of reconstructed images from MNIST, Fashion-MNIST, and K-MNIST datasets, illustrating variability in quality, including well-reconstructed (low MSE), near-average, and distorted (high MSE) cases.

Supplementary Table S1. Quantitative metrics (MSE, PSNR, and cosine distance) evaluating reconstruction performance of the autoencoder for MNIST, Fashion-MNIST, and K-MNIST.

Dataset \ Metrics	MSE*, m (σ)		PSNR†, m (σ)		cosine distance‡, m (σ)	
	Digital	Optical	Digital	Optical	Digital	Optical
MNIST	0.0236 (0.0115)	0.0295 (0.0151)	16.27 (2.11)	15.30 (2.19)	0.119 (0.0590)	0.518 (0.0734)
Fashion-MNIST	0.0352 (0.0193)	0.0449 (0.0252)	14.54 (2.38)	13.48 (2.42)	0.334 (0.166)	0.268 (0.108)
K-MNIST	0.0634 (0.0260)	0.0663 (0.0275)	11.98 (1.43)	11.79 (1.47)	0.595 (0.145)	0.481 (0.0732)

*$MSE = \frac{1}{n}\sum_{i=1}^{n}(x_i - x'_i)^2$. n: Total number of pixels in the image (e.g., for a 28×28 image, n = 784). x_i: Pixel value of the i-th pixel in the original image, x'_i: Pixel value of the i-th pixel in the reconstructed image.

†$PSNR = 10\log_{10}(\frac{MAX^2}{MSE})$. MAX: The maximum pixel value (1 for normalized images). MSE: The mean squared error.

‡$cosine\ distance = 1 - \frac{A \cdot B}{||A||\,||B||}$ A: Pixel values of the original image. B: Pixel values of the decoded image. Both A and B are normalized within the range [0,1].

Supplementary Table S2. Classification accuracy for 3,000 original test images and their reconstructions using digitally and optically implemented autoencoders.

Classification task	Original images (Test)	Reconstructed images (Digital)	Reconstructed images (Optical)
MNIST	95.1%	88.8%	85.1%
Fashion-MNIST	86.6%	68.4%	63.8%
K-MNIST	81.3%	69.0%	80.0%

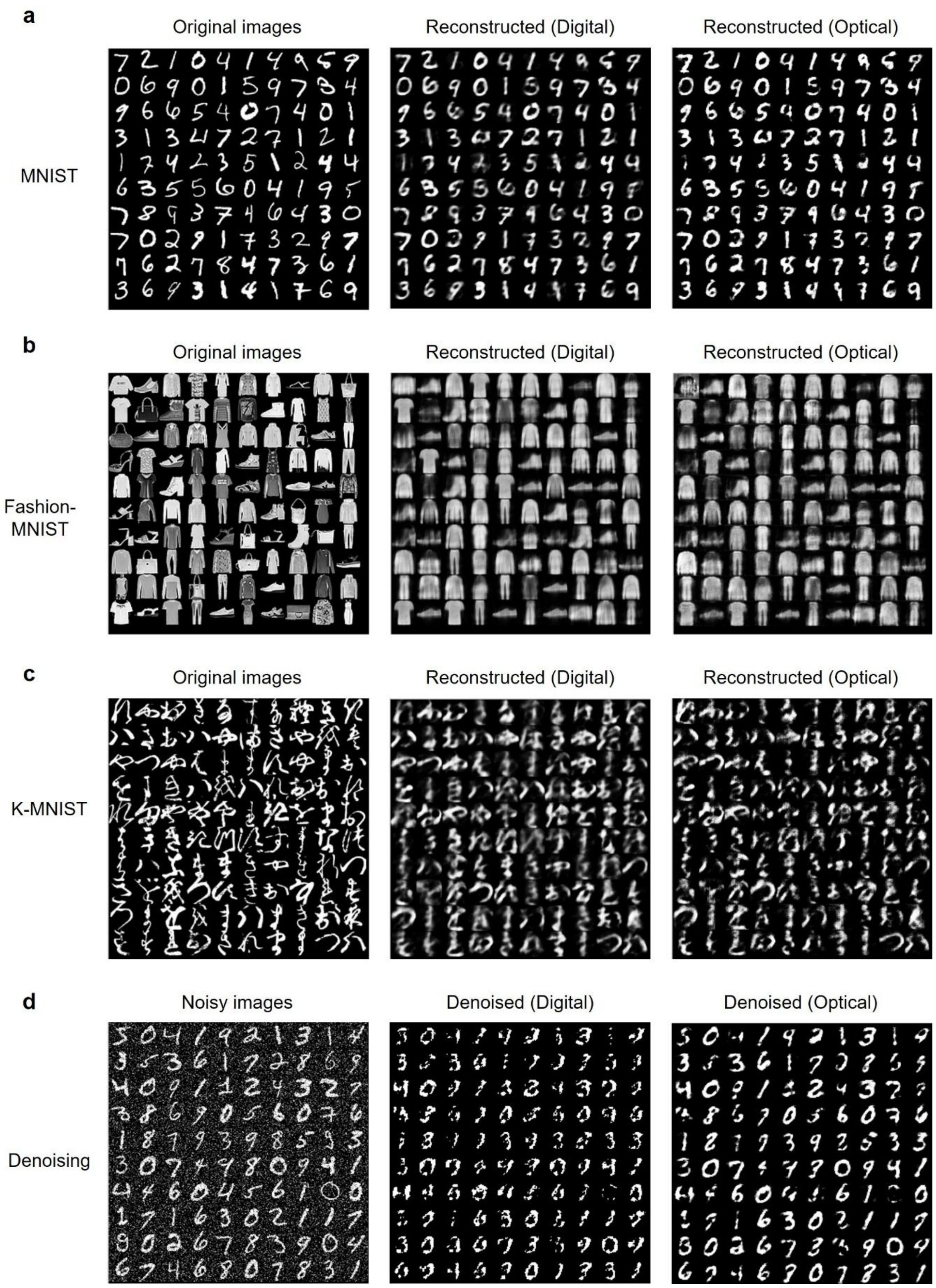

Fig. S7 Reconstructed images. a-c Representative examples of original images (left), reconstructed images with a digital autoencoder (middle), reconstructed images using our

optical multipliers (right) for MNIST (a), Fashion-MNIST (b), K-MNIST (c). **d** Representative examples of noisy input images (left), digitally denoised output images (middle), and output images denoised using our optical DAE (right).

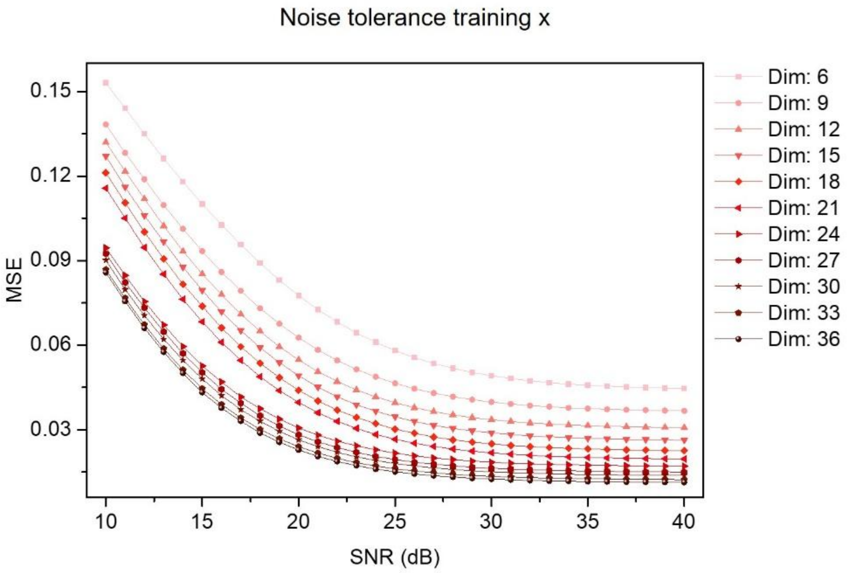

Fig. S8 MSE trends from models trained without built-in noise tolerance mechanisms, estimated for MNIST test data across varying SNR levels (bottom x-axis), with corresponding optical energy per pixel required for MVM operations (top x-axis).

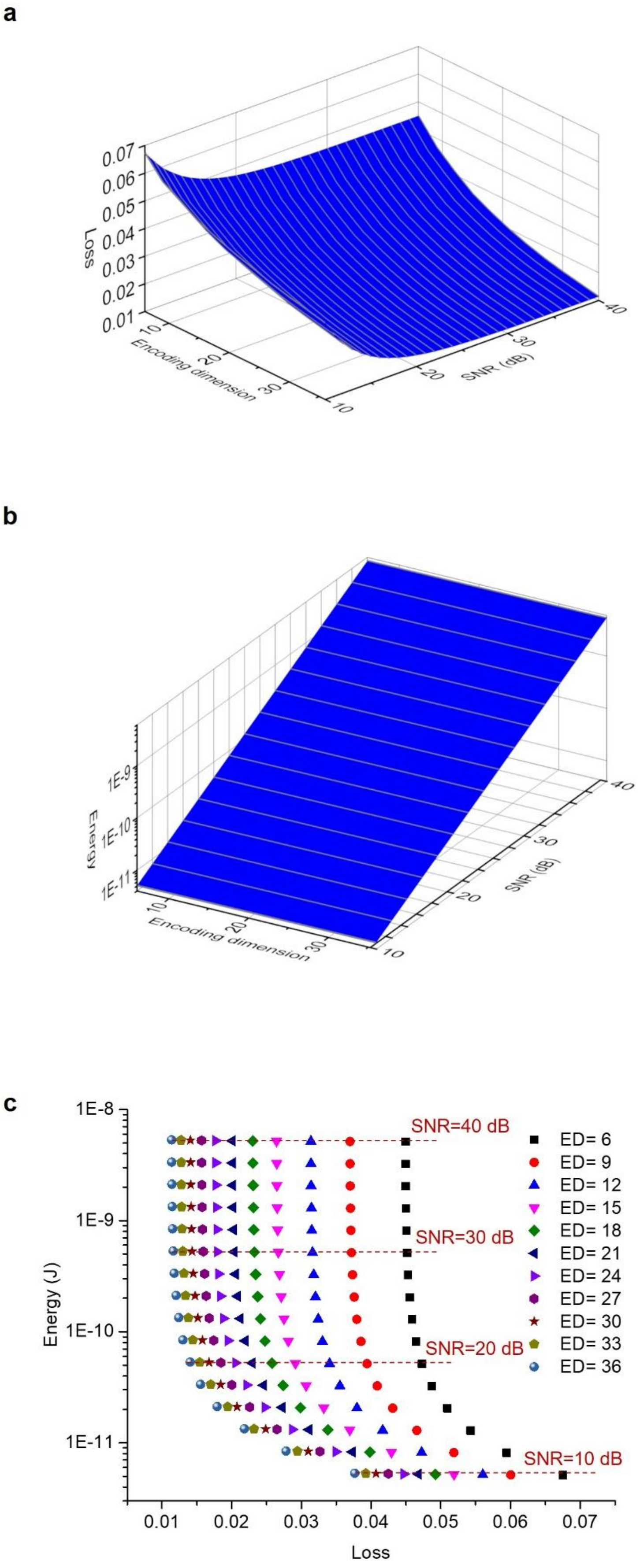
a
0.07
0.06
0.05
0.04
0.03
0.02
0.01
Loss
10
20
30
Encoding dimension
10
20
30
40
SNR (dB)
b
1E-9
1E-10
1E-11
Energy
10
20
30
Encoding dimension
10
20
30
40
SNR (dB)
c
1E-8
1E-9
1E-10
1E-11
Energy (J)
0.01
0.02
0.03
0.04
0.05
0.06
0.07
Loss
SNR=40 dB
SNR=30 dB
SNR=20 dB
SNR=10 dB
ED= 6
ED= 9
ED= 12
ED= 15
ED= 18
ED= 21
ED= 24
ED= 27
ED= 30
ED= 33
ED= 36

Fig. S9 Analysis of the relation among MSE loss, energy consumption, encoding dimension (ED) and signal-to-noise ratio (SNR). a 3D plot of MSE loss depending on the ED and SNR, showing that loss decreases as SNR increases and ED expands. **b** 3D plot of total optical energy used for encoding and decoding tasks as a function of ED and SNR, indicating that energy consumption increases with both expanding ED and rising SNR. **c** Relationship between MSE loss and optical energy consumption for varying ED. As ED increases, MSE loss decreases significantly, while energy consumption increases slightly due to higher encoding energy.

a

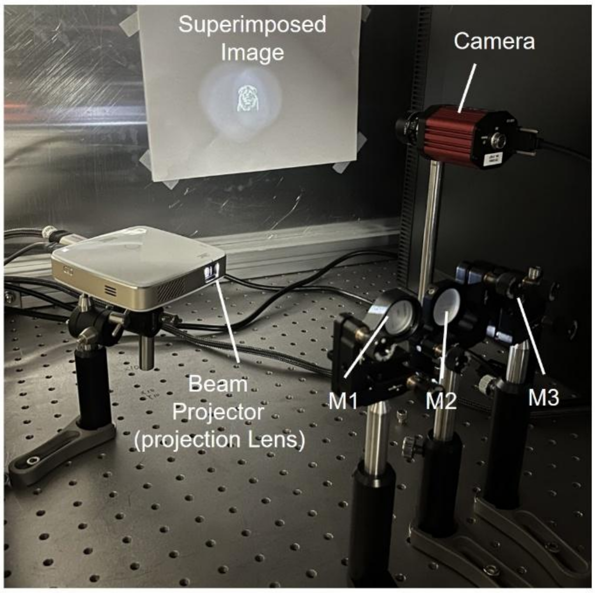

Superimposed Image
Camera
Beam Projector (projection Lens)
M1
M2
M3

b

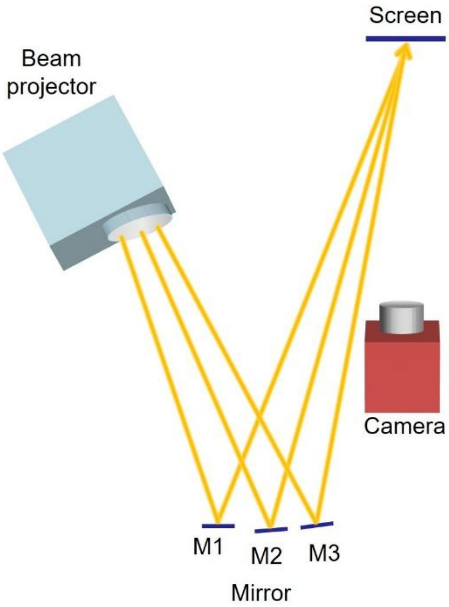

Screen
Beam projector
Camera
M1
M2
M3
Mirror

c

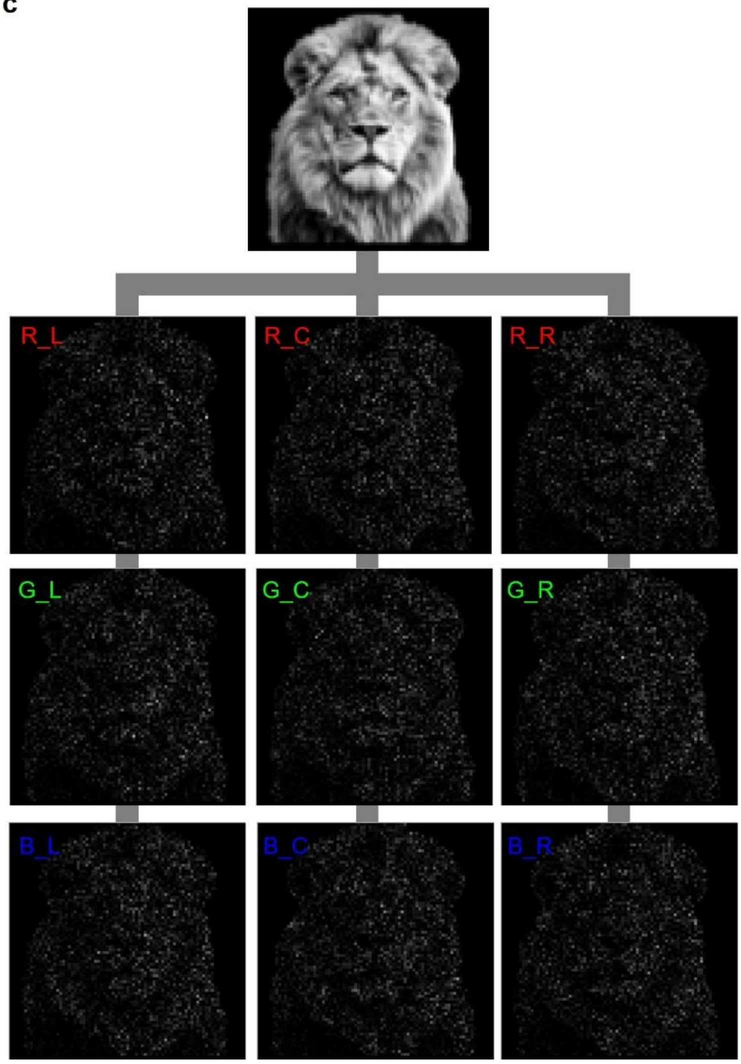

R_L
R_C
R_R
G_L
G_C
G_R
B_L
B_C
B_R

Fig. S10 Experimental demonstration of scalable image overlap using a beam projector and mirrors. a Photograph of the experimental setup, showing a beam projector equipped with projection lens, three mirrors (M1-M3), and a monochrome camera capturing the superimposed image. **b** Simplified schematic of the optical setup and beam path. **c** Example of splitting the original 80×80-pixel lion image into 9 sub-images, grouping them into sets of three, and encoding each set into the R, G, and B channels of a single color image, resulting in three sub-images per color image.

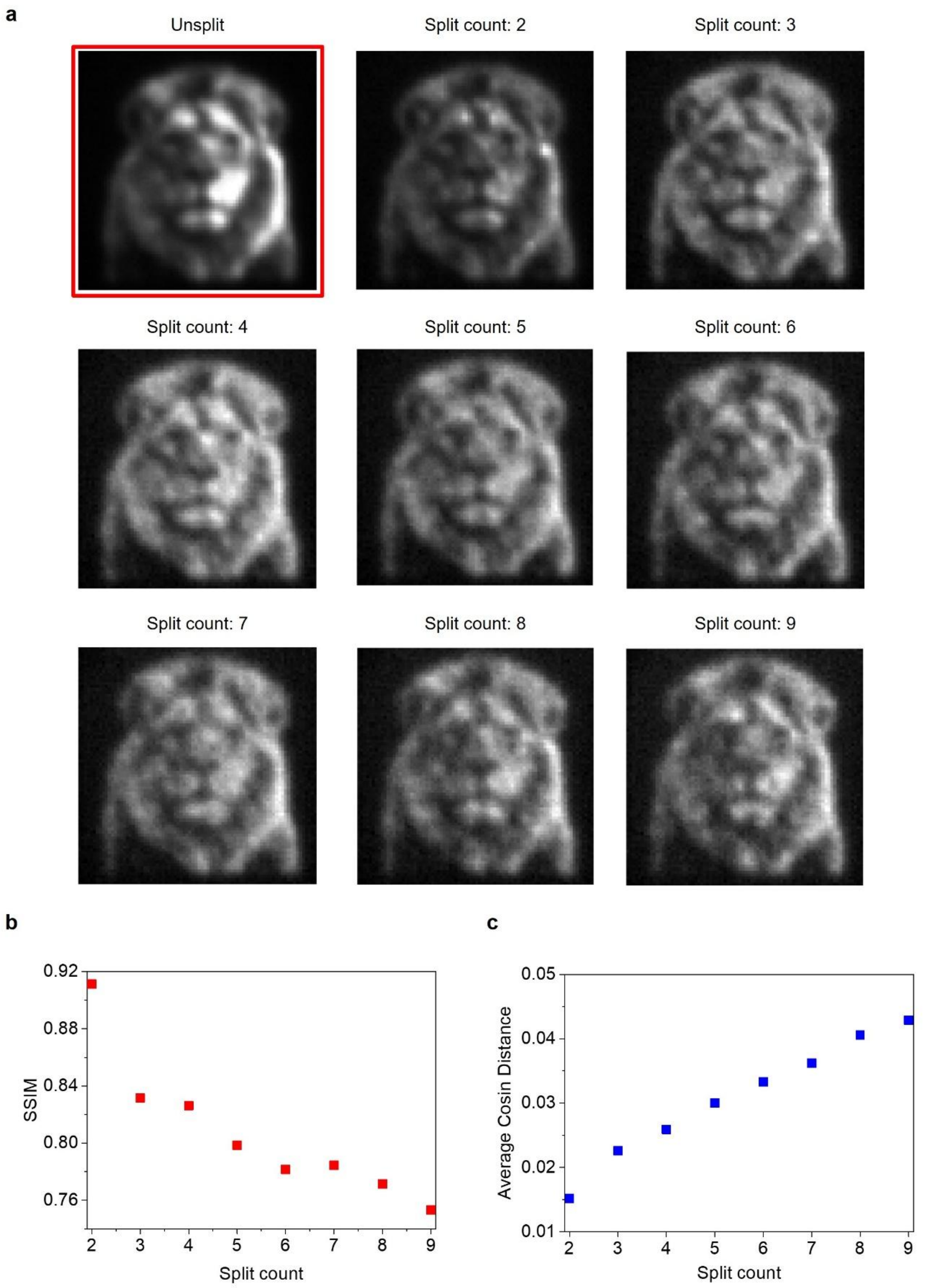

Fig. S11 Impact of pixel splitting and optical overlapping on image reconstruction. a Camera-captured images demonstrating the effect of splitting and overlapping pixels. The original unsplit 80×80-pixel lion image (highlighted by a red box) was directly captured by a

monochrome camera without pixel splitting. For the remaining images, sub-split images were generated for split counts ranging from 2 to 9, overlapped, and captured under identical conditions using the method described in Fig. S10. **b, c** Quantitative analysis showing the Structural Similarity Index (SSIM) and Cosine Distance, respectively, between the original unsplit image and each composite image as a function of split counts.

Supplementary References

1 Jeon, K. *et al.* Self-rectifying resistive memory in passive crossbar arrays. *Nature communications* **12**, 2968 (2021).

2 Song, C., Kim, J. & Jeong, D. S. Optimal Weight-Splitting in Resistive Random Access Memory-Based Computing-in-Memory Macros. *Advanced Intelligent Systems* **5**, 2200289 (2023).

3 Shen, Y. *et al.* Deep learning with coherent nanophotonic circuits. *Nature photonics* **11**, 441-446 (2017).

4 Lin, X. *et al.* All-optical machine learning using diffractive deep neural networks. *Science* **361**, 1004-1008 (2018).

5 Ryou, A. *et al.* Free-space optical neural network based on thermal atomic nonlinearity. *Photonics Research* **9**, B128-B134 (2021).

6 Glorot, X. & Bengio, Y. in *Proceedings of the thirteenth international conference on artificial intelligence and statistics.* 249-256 (JMLR Workshop and Conference Proceedings).

7 Wang, T. *et al.* Image sensing with multilayer nonlinear optical neural networks. *Nature Photonics* **17**, 408-415 (2023).

8 Hamerly, R., Bernstein, L., Sludds, A., Soljačić, M. & Englund, D. Large-scale optical neural networks based on photoelectric multiplication. *Physical Review X* **9**, 021032 (2019).
